# Preparation and Characterization of Chemically Cross-Linked Xanthan/Poly(Vinylalcohol) Hydrogel Films Containing Cerium Oxide Nanoparticles for Potential Application in Removal of Methylene Blue and Crystal Violet Dyes

**DOI:** 10.3390/gels11100809

**Published:** 2025-10-09

**Authors:** Nicusor Fifere, Maria Marinela Lazar, Irina Elena Raschip, Anton Airinei, Cristian-Dragos Varganici, Maria Valentina Dinu

**Affiliations:** “Petru Poni” Institute of Macromolecular Chemistry, Grigore Ghica Voda Alley 41A, 700487 Iasi, Romania; maria.lazar@icmpp.ro (M.M.L.); iecoj@icmpp.ro (I.E.R.); anton.airinei@icmpp.ro (A.A.); varganici.cristian@icmpp.ro (C.-D.V.); vdinu@icmpp.ro (M.V.D.)

**Keywords:** nanocomposite hydrogel, cross-linked films, CeO_2_NPs, morphology, optical properties, dye removal efficiency

## Abstract

In this work, hydrogel nanocomposites, as films, were prepared by embedding cerium oxide nanoparticles (CeO_2_NPs) within xanthan gum (Xn)/poly(vinylalcohol) (PVA) matrices. Their physicochemical properties were tuned by adjusting the ratio between components and thermal treatment conditions. The cross-linking of the polymer network was confirmed by attenuated total reflectance–Fourier transform infrared (ATR-FTIR), thermal analysis, and swelling behavior. Morphological features were evaluated by atomic force microscopy (AFM), scanning electron microscopy (SEM), while optical properties were investigated by UV–Vis spectroscopy. Undoped films displayed high transparency (~80% transmittance at 400 nm), with thermal cross-linking determined only slight yellowing and negligible changes in absorption edge (300 ± 2 nm). In contrast, CeO_2_NPs incorporation increased reflectance and introduced a new absorption threshold around 400 ± 2 nm, indicating nanoparticle–matrix interactions that modify optical behavior. Sorption studies with Methylene Blue (MB) and Crystal Violet (CV) dyes highlighted the influence of nanoparticle content and cross-linking on functional performance, with thermally treated samples showing the highest efficiency (~97–98% MB and 71–83% CV removal). Overall, the results demonstrate how structural tailoring and cross-linking control the characteristics of Xn/PVA/CeO_2_ nanocomposites, providing insight into their design as multifunctional hydrogel materials for environmental applications.

## 1. Introduction

In recent years, hydrogel nanocomposites (HNCs) have gained substantial attention as promising adsorbent materials for dye removal owing to their three-dimensional cross-linked polymeric networks, high water uptake capacity, tunable porosity, and ability to incorporate functional nanomaterials [1,2,3]. By combining the structural flexibility of hydrogels with the physicochemical activity of nanofillers, HNCs offer enhanced swelling behavior, stability, and adsorption efficiency compared to pristine hydrogels [2,4,5,6]. The performance of such hybrid systems strongly depends on the type of polymer matrix, cross-linking strategy, and incorporated nanoparticles.

Among the various inorganic nanofillers, metal oxide nanoparticles (NPs) such as TiO_2_ [7,8], Fe_3_O_4_ [9,10,11], SiO_2_ [12,13], and ZnO [14,15] have been extensively employed in HNCs for water purification applications [7,16,17]. Their nanoscale dimensions, high surface area, redox activity, and chemical stability impart unique adsorption, photocatalytic, and antimicrobial properties to the host hydrogel. For instance, Fe_3_O_4_-based hydrogels combine high adsorption with facile magnetic recovery [9,10,11], while TiO_2_- and ZnO-modified hydrogels [7,14] demonstrate enhanced photocatalytic degradation of dyes and their efficient removal. Several studies have reported remarkable improvements in MB adsorption capacity upon nanoparticle incorporation, with performance dominated by electrostatic interactions, ion exchange, and surface complexation [18,19]. However, challenges such as reduced swelling at high NP loadings, mechanical brittleness, and limited recyclability continue to motivate the search for novel formulations.

CeO_2_NPs are an emerging class of rare-earth oxides that have attracted significant attention owing to their unique redox-switching ability (Ce^3+^/Ce^4+^ transition), exceptional oxygen storage capacity, and remarkable chemical stability [20,21,22,23,24,25,26]. These properties underpin their use in diverse fields such as catalysis, sensing, biomedicine, and environmental remediation [20,21,22,23,24,25,26]. Nevertheless, the incorporation of CeO_2_NPs into hydrogel-based nanocomposites for wastewater treatment has not been investigated as extensively as other metal oxides. Embedding CeO_2_NPs within biopolymeric hydrogel networks could generate synergistic benefits, including rapid adsorption kinetics, enhanced structural durability, and improved recyclability under variable environmental conditions.

Xn and PVA are particularly promising candidates for fabricating such hybrid nanocomposites [27,28,29,30]. Xn, a natural, biodegradable polysaccharide, exhibits high hydrophilicity and possesses reactive functional groups suitable for chemical modification [28,31,32,33,34]. PVA, on the other hand, is a synthetic polymer well recognized for its film-forming ability, mechanical strength, and biocompatibility [34]. Through chemical cross-linking, Xn can form hydrogel films with tunable porosity and swelling behavior, providing an efficient platform for nanoparticle entrapment [35]. Combining these polymers offers a sustainable route to fabricate hydrogel nanocomposites that are eco-friendly, easy to process, and potentially scalable for large-scale water purification.

Previous research has demonstrated the versatility of Xn-based hydrogels in removing pollutants from aqueous systems. For example, Ghorai et al. [36] developed Xn-*g*-poly(acrylamide) copolymers and later functionalized them with silica nanoparticles for adsorption applications. Similarly, Xn-silica composites have been applied for the removal of Pb(II) [37], while other Xn-based nanocomposites have shown efficient adsorption of Cu(II), Pb(II), and Ni(II) ions [38]. More recent studies highlight Xn-derived hydrogels for dye adsorption, such as MB removal through Xn-maleic anhydride hydrogels [39], magnetic nanoparticle-modified Xn systems [40], and Xn-TiO_2_ hybrid hydrogels for binary and single dye removal [41].

While these studies confirm the high potential of Xn-based hydrogels for environmental applications, the entrapment of CeO_2_NPs within Xn/PVA hydrogel films remains unexplored. Recent developments have reported CeO_2_-based composites for other applications, such as cellulose/Xn/CeO_2_NPs films for active food packaging [42] and PVA/carboxymethyl cellulose/CeO_2_NPs hydrogels for antibacterial wound dressings [43]. These findings suggest that integrating CeO_2_ NPs into Xn/PVA hydrogel films could open new opportunities for designing multifunctional adsorbents with improved efficiency, stability, and reusability in wastewater treatment systems.

In this study, we report for the first time the preparation and characterization of chemically cross-linked Xn/PVA films entrapping CeO_2_NPs for some synthetic dyes from aqueous solutions, such as MB and CV. The films were synthesized through chemical cross-linking to ensure structural integrity and homogenous NP dispersion, followed by systematic characterization of their physicochemical, morphological, swelling, thermal, and optical properties. The sorption behavior of MB and CV onto the films was also investigated. The synergistic combination of Xn/PVA networks with CeO_2_NPs is expected to offer a novel, efficient, and recyclable adsorbent system, contributing to the development of advanced HNCs for dye-laden wastewater treatment.

## 2. Results and Discussion

### 2.1. Preparation of Xn/PVA/CeO_2_NPs Nanocomposite Films

**Figure 1:**
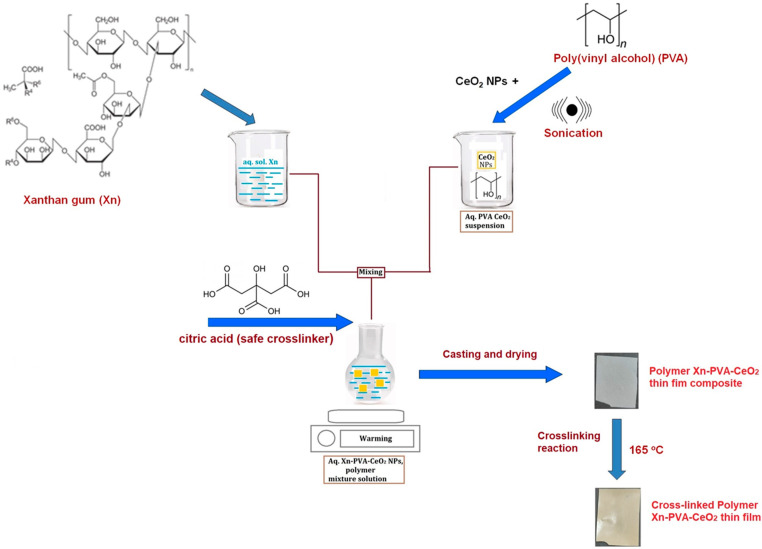
Schematic representation with the synthesis procedure of Xn/PVA/CeO_2_NPs films.

Nanocomposite hydrogel films based on Xn, PVA, and CeO_2_NPs were prepared using casting method with citric acid as a safe cross-linking agent (Figure 1). CeO_2_NPs were synthesized according to a previously published protocol [44]. The synthesized CeO_2_NPs showed very sharp and intense diffraction peaks located to the 2θ = 28.61°, 33.15°, 47.57°, 56.45°, 59.14°, 69.53°, 76.18°, and 79.19° (Figure S1). These correspond to cubic fluorite structure with the corresponding planes (111), (200), (220), (311), (222), (400), (331), and (420). The peaks shape and the absence of the foreign signals eliminate the possibility of cubic structure alteration by impurities or the presence of unwelcome phases [44]. The morphology revealed by TEM images show nearly rectangular shapes consisted of grains with random dimensions with average of about 17 nm [44] (Figure S2). The zero-point charge of CeO_2_NPs was also evaluated by streaming potential measurements as a function of pH and included as Figure S3 in Supplementary Materials.

**Figure 2:**
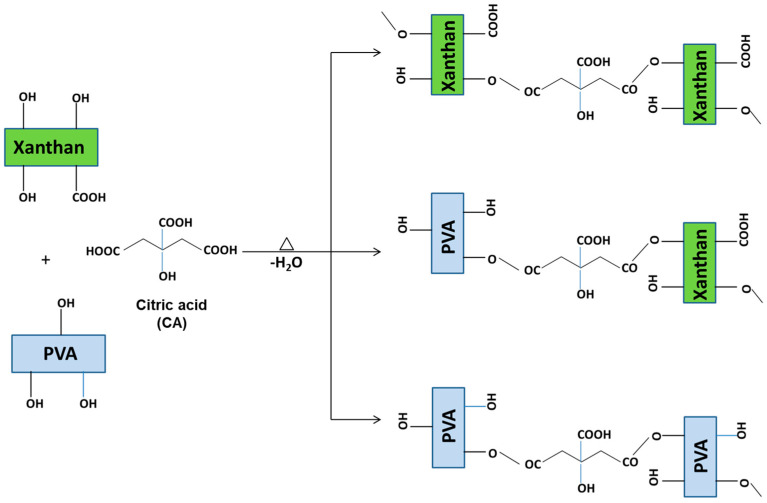
Schematic illustration of the CA–induced cross-linking process of Xn/PVA-based nanocomposite films.

To obtain nanocomposite hydrogels, Xn was first dissolved in water to obtain an aqueous solution, while PVA was dissolved separately and used to disperse CeO_2_NPs under sonication to ensure a homogeneous suspension (Figure 1). The Xn solution, PVA–CeO_2_NPs suspension, and citric acid (CA) were then combined to form a uniform polymer–nanoparticle mixture. This mixture was cast into films and dried to obtain Xn/PVA/CeO_2_ thin film composites. Finally, thermal treatment at 165 °C induced cross-linking through CA (Figure 2), yielding stable cross-linked Xn/PVA/CeO_2_ nanocomposite hydrogel films.

As Figure 2 shows, the cross-linking of Xn/PVA nanocomposite films is proceeds through esterification reactions promoted by CA under thermal treatment. Owing to its three carboxylic acid groups and one hydroxyl group, CA functions as a versatile cross-linker capable of forming covalent ester bonds with the hydroxyl groups of both Xn and PVA chains. During heating, partial water evaporation facilitates esterification and contributes to microstructural stabilization by reducing free water that could otherwise hinder cross-linking. Nonetheless, heating must be carefully controlled, as excessive dehydration may cause film brittleness, a phenomenon previously reported by Reddy and Yang for starch films cross-linked with CA [45], as well as in Xn-based hydrogels [46].

Table 1 summarizes the composition and thermal treatment conditions of the prepared samples. All formulations were based on a fixed Xn/PVA weight ratio of 50/50. The blank polymer matrix without nanoparticles is denoted as P0, while its thermally cross-linked counterpart treated at 165 °C is labeled P0-T. Nanocomposite films containing CeO_2_NPs were prepared with three different loading levels: 5% (P5), 10% (P10), and 15% (P15). For each composition, a corresponding thermally treated sample was also obtained at 165 °C (P5-T, P10-T, P15-T). Thus, the series of samples allowed for the systematic evaluation of both nanoparticle concentration and thermal cross-linking effects on the structural and optical properties of the Xn/PVA/CeO_2_ films.

### 2.2. Structural Characterization of Nanocomposite Hydrogel Films

The ATR-FTIR spectra provide insight into the chemical structure and cross-linking interactions within the prepared Xn/PVA/CeO_2_ nanocomposite films (Figures S4–S6, Supplementary Materials). In the ATR-FTIR spectra of the Xn/PVA hydrogel films, characteristic absorption bands of both Xn and PVA are observed. A broad band at ~3340–3400 cm^−1^ corresponds to O–H stretching vibrations of hydroxyl groups, while peaks at ~2920–2930 cm^−1^ arise from C–H stretching [33,34]. The band at ~1720 cm^−1^ is attributed to C=O stretching of carboxyl groups, and the region at ~1040–1070 cm^−1^ corresponds to C–O–C stretching of glycosidic linkages, confirming the polysaccharide backbone [33,34]. In addition, absorption in the ~1080–1140 cm^−1^ region is assigned to C–O stretching of secondary alcohols, also characteristic of PVA [33,34]. For the CeO_2_-containing nanocomposites, further spectral changes are evident. The O–H stretching band becomes broader, indicating enhanced hydrogen bonding interactions induced by nanoparticle incorporation. Slight shifts in the C=O and C–O–C bands suggest interactions between CeO_2_NPs and functional groups of the polymer matrix. Moreover, the new absorptions at ~627–650 cm^−1^ are assigned to Ce–O vibrations, confirming the successful incorporation of CeO_2_NPs [20].

Upon thermal treatment at 165 °C, the ester carbonyl absorption (~1720 cm^−1^) becomes more pronounced, demonstrating effective cross-linking by CA [45]. Simultaneously, a reduction in O–H stretching intensity supports the consumption of hydroxyl groups during esterification. These spectral changes confirm that CA efficiently cross-links the Xn/PVA matrix, while CeO_2_NPs remain well integrated within the polymer network. Similar trends are observed for all CeO_2_NPs containing nanocomposites (Figures S4–S6, Supplementary Materials).

**Figure 3:**
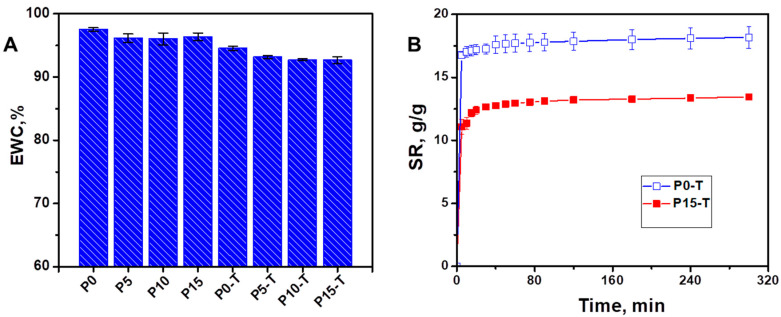
(**A**) Equilibrium water content (EWC) and (**B**) swelling ratio (SR) as a function of time of nanocomposite films.

Equilibrium water content (EWC) (Figure 3A) and swelling kinetics (swelling ratio, SR, as a function of time—Figure 3B) of the nanocomposite films were further evaluated in distilled water to prove cross-linking by CA. Untreated films (P0, P5, P10, P15) exhibited slightly higher EWC values (96.0–97.5%), with the maximum recorded for P0 (97.53 ± 0.28%). Upon CA cross-linking (P0-T, P5-T, P10-T, P15-T), a moderate but consistent reduction in EWC was observed, with values decreasing to 94.55 ± 0.32% (P0-T) and further to 92.66 ± 0.57% (P15-T). The decrease in EWC with increasing CeO_2_NPs content after cross-linking suggests that the denser three-dimensional network formed by esterification restricted the free volume available for water uptake.

The SR profiles further highlighted the influence of CA cross-linking (Figure 3B). Both P0-T and P15-T reached equilibrium within ~300 min; however, their swelling capacities were significantly different. P0-T exhibited the highest swelling ratio (~17–18 g/g), while P15-T stabilized at ~13–14 g/g. The lower SR of P15-T compared to P0-T indicates that the incorporation of CeO_2_NPs combined with cross-linking, generated a tighter network with increased cross-link density. This restricted chain mobility and water penetration, resulting in reduced swelling.

**Figure 4:**
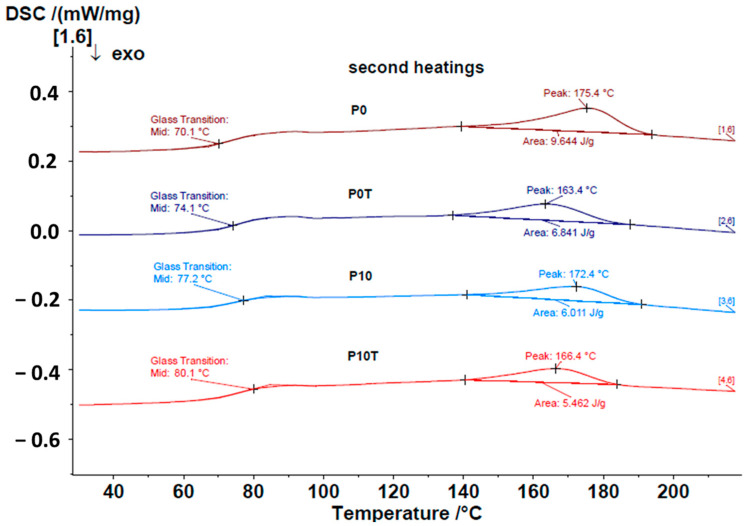
DSC curves for P0, P0-T, P10 and P10-T nanocomposite films.

In addition, differential scanning calorimetry (DSC) was performed to evaluate the influence of CeO_2_NPs on the Xn/PVA polymer matrix before and after chemical cross-linking. The first heating curves were used to clear the thermal history, and thus the relevant thermal transitions were extracted from the cooling and secondary heating scans (Figure 4).

For the composite films without nanoparticles, an increase in glass transition temperature (T_g_) is observed upon cross-linking: from 70.1 °C for the uncross-linked sample (P0) to 74.1 °C for the cross-linked sample (P0-T). This shift is consistent with the restricted mobility of polymer chains after cross-linking, which requires more thermal energy to enable chain motion. At the same time, the melting temperature (T_m_) decreases significantly, from 175.4 °C for P0 to 163.4 °C for P0-T. This reduction indicates that cross-linking hinders chain mobility and reduces crystallinity, thereby promoting a more amorphous structure. When CeO_2_NPs are incorporated into the polymer matrix, T_g_ increases further. The uncross-linked nanocomposite (P10) shows a T_g_ of 77.2 °C, higher than the corresponding neat polymer (P0). This increase demonstrates an interaction between the nanoparticles and the polymer chains, which restricts their mobility. Upon cross-linking, the T_g_ of the P10-T sample rises to 80.1 °C, the highest among all tested systems. This result suggests enhanced interactions between CeO_2_NPs and the cross-linked polymer network. Regarding melting transitions, both uncross-linked samples (P0 and P10) exhibit similar T_m_ values (175.4 °C and 172.4 °C, respectively). After cross-linking, both P0-T and P10-T show decreased melting temperatures (163.4 °C and 166.4 °C, respectively). The comparable reduction in T_m_ upon cross-linking indicates that CeO_2_NPs incorporation does not significantly affect the crystalline character of the polymer matrix.

### 2.3. Morphology of Nanocomposite Hydrogel Films

**Figure 5:**
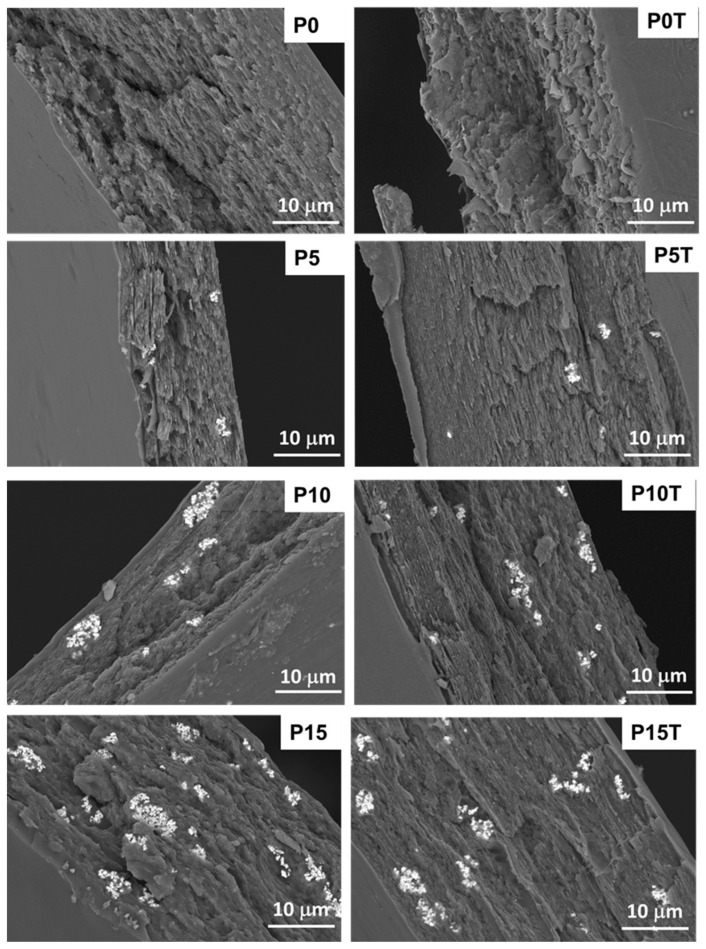
SEM micrographs of cross-sections through the nanocomposite hydrogel films untreated (P0, P5, P10, and P15) and thermal treated at 165 °C (P0-T, P5-T, P10-T, and P15-T).

The SEM micrographs reveal distinct morphological differences between the neat polymer matrix and the CeO_2_-loaded nanocomposite films, as well as between untreated and thermally cross-linked samples (Figure 5).

The blank polymer film (P0) displays a relatively rough and irregular fracture surface, indicating a brittle fracture behavior, while the thermally treated sample (P0-T) shows a more compact and homogeneous morphology, reflecting improved cohesion due to cross-linking.

Upon incorporation of CeO_2_NPs, the film surface become rougher, and bright regions corresponding to CeO_2_NPs aggregates are clearly visible. At low loading (P5), the nanoparticles are sparsely and relatively homogeneously distributed, with only minor aggregation. Increasing nanoparticle content to 10% (P10) results in more pronounced clusters embedded within the polymer matrix, while at the highest concentration (P15), the agglomeration of CeO_2_NPs is evident (Figure 5).

Thermal treatment of the nanocomposite films (P5-T, P10-T, P15-T) produces denser and more compact surfaces compared to the untreated samples (Figure 5). The improved adhesion between the polymer matrix and CeO_2_NPs is suggested by the tighter embedding of nanoparticle clusters within the cross-linked network. However, at high loading (P15-T), although cross-linking enhances matrix integrity, significant nanoparticle agglomerates remain visible (Figure 5). The EDX analysis provides compositional confirmation that CeO_2_NPs are effectively incorporated within the Xn/PVA polymer matrix (Figure S7 and Table S1, Supplementary Materials). The increasing intensity of Ce signals with higher nanoparticle loading directly supports the morphological observations from SEM. The EDX results not only confirm the presence of Ce but also reveal a systematic increase in its relative amount with nanoparticle content (Figure S7 and Table S1, Supplementary Materials).

**Figure 6:**
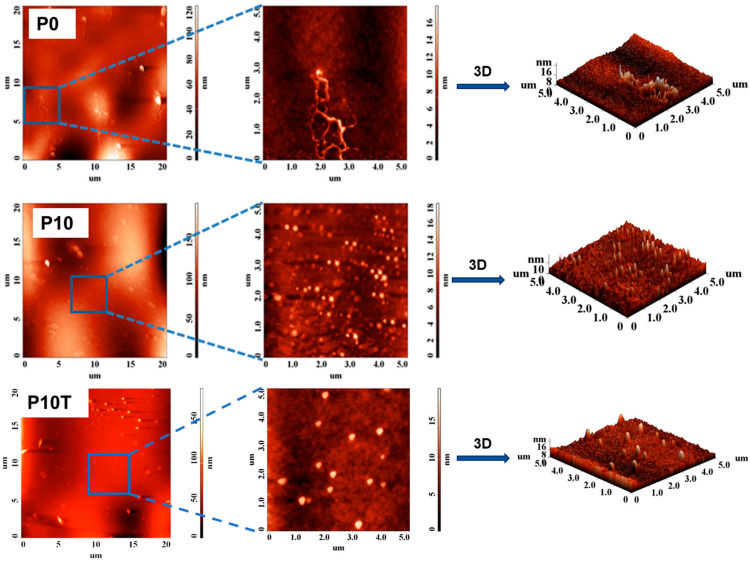
AFM topographic images of Xn/PVA/CeO_2_ nanocomposite hydrogel films: P0 (without CeO_2_NPs), P10 (10% CeO_2_NPs), and P10T (10% CeO_2_NPs with thermal treatment). Each sample is shown at different scan sizes: 20 × 20 µm^2^ (**left**), 5 × 5 µm^2^ zoomed regions (**middle**), and corresponding 3D topographic reconstructions (**right**).

AFM analysis was also employed to investigate the surface morphology of Xn/PVA/CeO_2_ hydrogel nanocomposites (Figure 6).

The untreated sample without CeO_2_NPs (P0) exhibited relatively smooth surfaces with a few irregular protrusions, as evidenced by the lower root mean square (RMS) roughness values (1.473–9.401 nm). Upon the incorporation of CeO_2_NPs (P10), the surface became rougher, with well-dispersed nanoparticle-like domains clearly visible at the nanoscale. This was further reflected in the RMS roughness values, which increased to 1.822 nm at 5 × 5 µm^2^ and reached 24.367 nm at 20 × 20 µm^2^ (Table 2), indicating significant microstructural heterogeneity.

After thermal treatment (P10-T), the films displayed a more uniformly textured surface with spherical protrusions corresponding to nanoparticle aggregates. Notably, the RMS roughness further increased to 2.142 nm (5 × 5 µm^2^) and 31.987 nm (20 × 20 µm^2^), suggesting thermally induced reorganization of the polymer matrix and nanoparticle domains. The synergistic effect of CeO_2_ loading and cross-linking [35] is therefore responsible for the enhanced nanoscale texturing, which is likely to influence key physicochemical properties such as water uptake, chain mobility, thermal stability, and optical response.

### 2.4. Optical Analysis

#### 2.4.1. Optical Properties of Hydrogel Films Without CeO_2_NPs

The initial optical characterization was performed on non-doped polymeric films to compare the polymer matrix in its untreated state (P0) with the thermally aged matrix (165 °C for 7 min, referred to as P0-T) (Figure S8). Both untreated and thermally aged films exhibit very low reflectivity (Figure S8a). However, this low reflectance is associated with the high transparency of the thin film rather than absorption. This is confirmed by the transmission spectra (Figure S8b), where transmittance reaches ~80% at 400 nm. Upon thermal treatment (165 °C, 7 min), the reflectance spectra decrease further, with noticeable changes in spectral shape up to 400 nm (Figure S8a). The corresponding transmission spectra (Figure S8b) show similar spectral modifications but without significant reduction in transparency. This suggests that the decrease in reflectance after thermal cross-linking is mainly due to a slight reduction in film brightness. Visually, the initially colorless transparent film becomes faintly yellow, though it retains nearly the same transparency. The inflection points in the reflectance and transmission spectra obtained from the first derivative corresponds to the absorption threshold. For both untreated and thermally treated undoped films, the transparency limit appears at 300 ± 2 nm. Therefore, thermal cross-linking does not significantly alter the optical properties of the polymer matrix in the absence of nanoparticles.

#### 2.4.2. Optical Properties of Nanoparticle-Doped Films

Xn-based films doped with CeO_2_NPs exhibit a pronounced increase in reflectance with increasing nanoparticle concentration, both for untreated (P5, P10, P15) and thermally treated (P5-T, P10-T, P15-T) nanocomposites (Figure S9a,c). This behavior is attributed to refractive index mismatch at the nanoparticle–polymer interface [47]. The interfacial area, between the two components, increase with nanoparticle content, leading to a corresponding increase of diffuse reflected light. The level of transparency instead, increases in reverse order, by decreasing the doping level (Figure S9b,d), due to the amount of reflected light that is getting weaker. It is important to note that the transmission spectra shown in Figure S9b,d have the first derivative with maximum located around wave length 300 nm ± 2 nm, maintaining the transparency limit at the same wavelength as the non-doped matrix, regardless of the nanoparticle concentration (Table S2, Supplementary Materials). On the other hand, the maximum values of the first derivative of the diffuse reflectance spectra, of the sample that contain CeO_2_NPs, shows an absorption threshold, around 400 nm ± 2 nm, as can be seen in Table S2, Supplementary Materials. Therefore, in that case a new optical transition occurs that clearly corresponds to the presence of the CeO_2_NPs in polymer matrix, which can be highlighted only by diffuse reflection measurements. Through the thermal treatment at 165 °C, the optical threshold, obtained from the first derivative of the reflectance spectrum, remains almost unchanged even if the shape of the spectrum was slightly changed.

Analysis of the derivative form of the Tauc relation [47,48,49,50] (Figure S10, Supplementary Materials), applied to CeO_2_NPs deposited on quartz glass, clearly indicates *n* = 0.5, confirming a direct allowed transition. The Tauc representation of the reflectance spectra, expressed as FR∞E1n=fE, for CeO_2_NPs is shown in Figure S11, Supplementary Materials.

#### 2.4.3. Effect of Doping and Thermal Treatment

In polymer films doped with CeO_2_NPs without thermal treatment, two linear regions in the Tauc representation are preserved only for the 5% (P5) and 10% (P10) doping levels, for both direct and indirect transitions (Figure S12a–d, Supplementary Materials). The corresponding bandgap energies, calculated for both types of transitions, are listed in Table 3. These values show no significant differences compared to those obtained for bare CeO_2_NPs. For the 15% doped sample (P15), also without thermal treatment, the modified Tauc relation exhibits linearity in only a single region, regardless of the transition type (Figure S12e,f, Supplementary Materials). The bandgap energies calculated for both direct and indirect transitions (Table 3) are below 4 eV, and are close to the sub-band transition values observed in bare CeO_2_NPs [51,52,53,54].

Thermal treatment induces polymer cross-linking, resulting in films where the Tauc representation exhibits linearity within a single domain for P5-T, P10-T, and P15-T samples (Figure S13, Supplementary Materials). This behavior corresponds to sub-band transitions [55,56,57,58,59], characterized by bandgap energies below 4 eV (Table 3).

The change in the optical response of the doped polymer films—compared with bare CeO_2_NPs could be attributed to the interaction between the nanoparticles and the polymer matrix. This effect becomes evident with increasing nanoparticle concentration (15% in the P15 sample, without thermal treatment) and in all thermally cross-linked films (P5-T, P10-T, and P15-T).

Similar modifications of optical transitions due to nanoparticle–matrix interactions have been reported in other systems, such as CeO_2_–PVA composites [60]. Moreover, studies on other semiconductors [61] have demonstrated that interactions with the supporting substrate can lead to the formation of interfacial species. These generate local defects that introduce extended localized states within the bandgap, thereby altering the optical properties. Such defect-induced localized states can make sub-band optical transitions predominant, limiting the applicability of the Tauc method for determining the interband transition energy (~4 eV). Consequently, the transition from two linear regions in the Tauc representation—observed for bare CeO_2_ nanoparticles—to a single linear region in doped polymer films serves as evidence of strong nanoparticle–matrix interactions in the Xn/PVA host. Experimentally, these interactions are observed both in the untreated P15 sample and in all thermally cross-linked films containing CeO_2_NPs.

The interaction between CeO_2_NPs and the Xn-based polymer can induce structural and morphological modifications, which are expected to increase in intensity with the density of nanoparticle–polymer interactions. Furthermore, thermal cross-linking may promote additional structural disorder across the material, generating local defects in the arrangement of macromolecular chains. Such changes directly influence the optical properties by introducing localized states within the bandgap region [62].

#### 2.4.4. Urbach Energy Analysis

The Urbach energy is widely used in the literature as a parameter proportional to the degree of structural disorder [63,64]. The values of $E_{U}$ were calculated from the reflectance spectra using the reciprocal of the slope in the linear representation of Equation (1), and are reported in Table 3. For the non-thermally treated samples, the polymer matrix alone exhibits significantly higher Urbach energy values compared with the composite films containing CeO_2_NPs. Furthermore, the Urbach energy decreases with increasing nanoparticle concentration in the polymer matrix, following the order 5% > 10% > 15% for non-cross-linked samples. This trend indicates a tendency toward structural ordering in the non-thermally treated composites, both upon nanoparticle incorporation and with increasing nanoparticle content. However, starting from 10% nanoparticle loading (P10) and continuing at 15% (P15), the changes in Urbach energy become less pronounced, suggesting a relative stabilization of the structure at higher doping levels.

In contrast, thermal cross-linking results in an increase in Urbach energy for all doping levels, indicating enhanced disorder, as previously discussed. The relative increase in Urbach energy for the thermally treated films ($E_{U,t})$, P5-T, P10-T, P15-T compared to their untreated counterparts ($E_{U,u})$ for the samples P5, P10, P15, expressed as *ΔE_U_*, is given in Table 3. These values were calculated for every pair of samples, P5-T, P5; P10-T, P10; P15-T, P15 with the Equation (1):

(1) $$∆E_{U}=\left(\frac{E_{U,t}}{E_{U,u}}-1\right)100$$

The highest increase is observed for the 5% doped sample ($∆E_{U}=94.85\%)$, while significantly lower values are obtained for the 10% and 15% doped samples ($∆E_{U}=54.27\%$ and 52.65%, respectively). Thus, the 5% doped sample shows the largest increase in disorder after thermal cross-linking, with a pronounced rise in Urbach energy. Interestingly, even the introduction of 5% nanoparticles in the polymer matrix leads to a slight increase in disorder upon thermal treatment compared to the undoped sample. By contrast, at 10% and 15% doping levels, nanoparticle incorporation appears to stabilize the degree of disorder following thermal cross-linking, as evidenced by the relatively constant ΔE_U_ values (~54–53%).

### 2.5. Sorption of Synthetic Dyes

Synthetic dyes are among the most widely used chemicals in modern industries such as textiles, paper, plastics, leather, cosmetics, and food processing. Their widespread application has resulted in the discharge of large volumes of dye-containing effluents into aquatic ecosystems [14,16,39]. Even at very low concentrations, synthetic dyes such as MB and CV are highly visible, imparting strong coloration to water bodies and reducing light penetration. This interferes with aquatic photosynthesis and disrupts food chains. Moreover, many dyes and their degradation products are toxic, mutagenic, or carcinogenic, and can cause skin irritation, respiratory distress, and organ dysfunction in humans [39]. Their recalcitrant nature and potential to generate hazardous by-products during conventional treatment processes (e.g., chlorination) make their removal particularly challenging.

**Figure 7:**
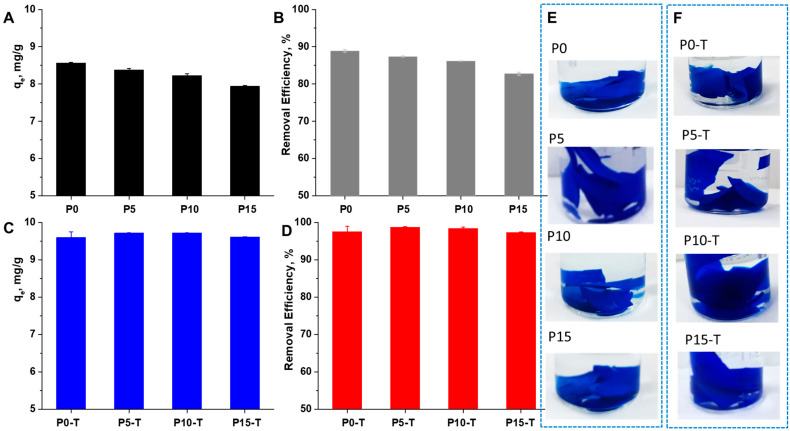
MB removal efficiency of Xn/PVA/CeO_2_ nanocomposite hydrogels: (**A**) The equilibrium adsorption capacity (q_e_, mg/g) and (**B**) MB removal efficiency (%) of non-thermally treated samples, (**C**) The equilibrium adsorption capacity (q_e_, mg/g) and (**D**) MB removal efficiency (%) of thermally treated samples, and (**D**–**F**) representative photographs of hydrogel samples after MB adsorption (P0, P5, P10, P15 (**E**) and their thermally treated counterparts (**F**)).

Given these concerns, the development of effective, sustainable, and low-cost technologies for dye removal from wastewater has become an urgent global priority. Adsorption-based methods, particularly those employing polymer-based hydrogels reinforced with functional nanomaterials, have emerged as promising candidates due to their high efficiency, environmental friendliness, and ability to be tailored for specific pollutants [14,16,36,37,38,39,41]. In this context, the MB removal efficiency of the Xn/PVA/CeO_2_ nanocomposite hydrogels was systematically evaluated to determine the effect of nanoparticle incorporation and thermal treatment (Figure 7).

**Figure 8:**
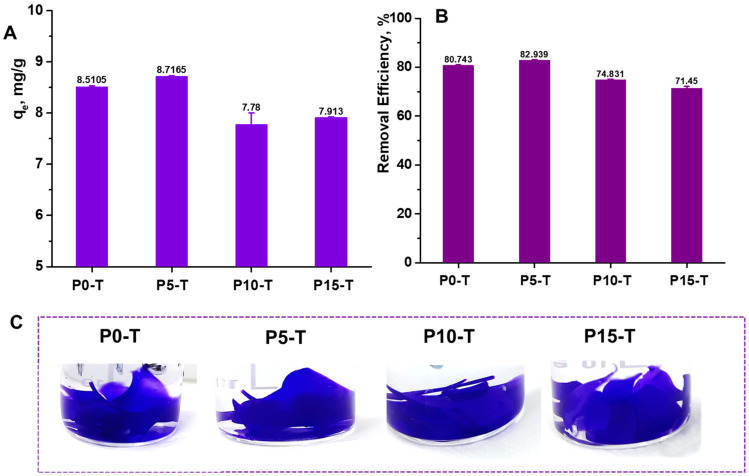
CV removal efficiency of thermally treated Xn/PVA/CeO_2_ nanocomposite hydrogels: (**A**) The equilibrium adsorption capacity (q_e_, mg/g) and (**B**) CV removal efficiency (%) for P0-T, P5-T, P10-T, and P15-T samples, and (**C**) representative photographs of hydrogels after CV adsorption.

As shown Figure 7, all samples demonstrated efficient dye adsorption capacity, confirming the affinity of the hydrogel matrix toward cationic dye molecules. The non-thermally treated samples (blue bars), including P0, P5, P10, and P15, exhibited relatively similar MB removal efficiencies, indicating that the initial incorporation of CeO_2_NPs did not markedly alter the adsorption capacity at this stage. This suggests that in the untreated state, the adsorption sites within the polymeric network remain comparable regardless of nanoparticle loading. In contrast, the thermally treated samples (red bars) displayed consistently higher MB removal compared to their untreated counterparts. This enhancement can be attributed to the structural reorganization of the polymer matrix upon thermal treatment, which increases porosity and surface roughness, as corroborated by the AFM analysis. The improved accessibility of active sites, along with the potential catalytic contribution of CeO_2_NPs, likely contributes to the higher adsorption efficiency. The control comparison (gray bars) further confirms that the presence of CeO_2_ alone contributes positively, but the synergistic effect of CeO_2_ loading combined with thermal treatment leads to the best performance. Among the samples, P5T and P10T exhibited the highest MB removal (Figure 8), highlighting the beneficial role of optimized nanoparticle concentration coupled with post-treatment in creating a more effective adsorbent material. Optical images of the hydrogels after MB adsorption provide visual confirmation of the quantitative results. The treated samples show more intense color uptake, indicating higher retention of MB molecules within the hydrogel matrix.

CV removal studies further highlight the role of CeO_2_NPs incorporation and thermal treatment in enhancing dye adsorption performance (Figure 8). The thermally treated pure hydrogel (P0-T) exhibited a moderate removal capacity, indicating that the polymeric matrix alone provides some accessible adsorption sites. With the inclusion of CeO_2_NPs, removal efficiency improved, but the performance was strongly dependent on the nanoparticle concentration.

At 5% loading (P5-T), the hydrogel demonstrated the highest CV removal (~83%), which can be attributed to the presence of CeO_2_NPs that may contribute through electrostatic interactions and surface complexation with dye molecules. However, at higher loading (P15-T), the removal efficiency slightly decreased, most likely due to nanoparticle agglomeration, which reduces the effective surface area and obstructs the diffusion of dye molecules into the polymeric network. These findings suggest that an optimal nanoparticle concentration (5% CeO_2_) maximizes dye adsorption efficiency, while excessive loading may hinder performance.

In Table 4 are provided a comparative overview of the removal efficiencies of various sorbents reported in the literature [8,9,10,12,18,39,41], benchmarked against the performance of the nanocomposite hydrogels developed in this study.

Most sorbents exhibited removal efficiencies for MB, typically ranging from ~63% to ~98%, depending on the type of polymer matrix, nanoparticle incorporation, and experimental conditions. For instance, TiO_2_-containing guar gum and cross-linked acrylated Xn-based hydrogels demonstrated removal efficiencies above 93%, while CMC-*g*-PAAm/CL-Fe_3_O_4_ achieved ~95% under alkaline pH. Similarly, TiO_2_-containing gum ghatti -*cl*-PAAm hydrogels showed excellent adsorption performance (~98%). In contrast, the removal of CV generally appeared more challenging, with efficiencies often lower than those observed for MB. For example, HPAM/SiO_2_@Xn composites reached ~95%, while the Xn-*cl*-2-(N-morpholinoethyl methacrylate)/TiO_2_ hydrogels removed only ~60% of CV under strongly alkaline conditions.

In comparison, the Xn/PVA/CeO_2_ nanocomposite hydrogels developed in this study demonstrated competitive performance, achieving ~97–98% removal of MB at pH 5.8, with a relatively low sorbent dosage (5 g/L). Although the removal efficiency for CV was slightly lower (~71–83%), it is still notable considering the mild operating conditions and the structural stability of the cross-linked hydrogel films.

Desorption of MB and CV dyes from the Xn/PVA/CeO_2_ nanocomposite hydrogels was accomplished using a two-step chemical regeneration strategy that combined acidic/organic and alkaline treatments. This sequential approach enabled efficient desorption, achieving 95% recovery for MB and complete (100%) recovery for CV, while simultaneously restoring the integrity of the cross-linked nanocomposite films for reuse in subsequent adsorption cycles. Notably, only the chemically cross-linked hydrogels withstood the regeneration process and fully retained their structural stability, whereas the uncross-linked counterparts were irreversibly degraded after alkaline treatment (see Figure S14). Future investigations will focus on the application of these chemically cross-linked nanocomposite hydrogels for systematic dye removal studies, including the effects of pH, initial dye concentration, temperature, contact time, and performance across multiple sorption–desorption cycles.

## 3. Conclusions

In this study, Xn/PVA/CeO_2_ nanocomposite hydrogel films were successfully developed via citric acid-mediated thermal cross-linking, achieving effective structural integration, enhanced surface morphology, and tunable optical properties. FTIR confirmed esterification-based cross-linking and CeO_2_ incorporation, while SEM and AFM showed improved surface roughness, compactness, and nanoparticle–polymer interactions, with P10T exhibiting the optimal balance. The addition of CeO_2_ introduced a new absorption threshold at ~400 nm while maintaining transparency above 300 nm. All films demonstrated appreciable adsorption of methylene blue and crystal violet, with thermal treatment enhancing performance through increased porosity and active site accessibility; moderate CeO_2_ loading (P5T and P10T) yielded the highest dye removal, whereas excessive loading reduced efficiency due to agglomeration. These results highlight the potential of controlled CeO_2_ incorporation and thermal cross-linking to produce hydrogels with superior structural, optical, and adsorption properties. Future work should explore hydrogel recyclability, scale-up synthesis, and performance in real industrial wastewater, aiming to optimize efficiency, mechanical integrity, and sustainability for practical wastewater treatment applications.

## 4. Materials and Methods

### 4.1. Materials

Xn and PVA were acquired from Sigma-Aldrich Chemie GmbH (Schnelldorf, Germany) and employed in nanocomposite film preparation without further purification. The average molecular weight of Xn (Mv = 1.98 × 10^6^ g·mol^−1^) was determined by viscometric analysis, following the method described by Pelletier et al. [65]. The number-average molar mass and polydispersity index of PVA (Mn = 96,619 g mol^−1^; PDI = 1.17) were determined by gel permeation chromatography. CeO_2_NPs were obtained following a previously established protocol [44]. CA (≥99.5%), methanol (≥99.9%), and hydrochloric acid (37%, *v*/*v*) were provided by Chemical Company (Iasi, Romania), while sodium hydroxide (≥98.0%) was purchased from Fluka Chemie GmbH (Buchs, Switzerland). all reagents were used without additional purification. Deionized water was used throughout the experiments.

### 4.2. Preparation of Polymeric Films

A series of polymeric films were developed from aqueous blends containing Xn and PVA in equal proportions, citric acid as a cross-linking agent, and CeO_2_NPs as filler. The films were prepared by solution casting, followed by controlled drying and thermal treatment. For each formulation, Xn and PVA were used in equal amounts, each contributing 50% of the total dry polymer mass. CA was added as a cross-linker at a ratio of 1 to 20 relative to the total dry polymer mass. CeO_2_NPs were incorporated at three different concentrations, specifically 5%, 10%, and 15% by weight, relative to the total polymer content. A control sample without nanoparticles was also prepared for comparative analysis.

For synthesis, aqueous solutions of Xn and PVA were fist prepared separately, each at a concentration of 1% (*w*/*v*). For each film, 20 g of 1% Xn solution and 20 g of 1% PVA solution were measured to give a total of 40 g of polymer solution. This volume was selected to occupy approximately half the volume of the Teflon Petri dish used for casting, ensuring uniform film thickness.

The incorporation of CeO_2_NPs was carried out in the PVA solution. A 20-g aliquot of the 1% PVA solution was transferred to a 100 mL round-bottom flask equipped with two necks. The appropriate amount of CeO_2_NPs (calculated based on 5%, 10%, or 15% of the polymer dry mass) was added to the solution. The suspension was subjected to ultrasonication and to a stirring speed of 700–800 rpm for 30 min to one hour to promote uniform dispersion of the nanoparticles. CA was then added to the PVA–nanoparticle suspension in an amount corresponding to 1/20 of the total dry polymer mass, which equated to 0.02 g in all cases. The mixture was heated at 80 °C using a preheated water bath under reflux conditions to prevent evaporation. Stirring was maintained vigorously for 20 min.

Following this step, 20 g of the 1% Xn solution were added directly to the warm mixture, and the entire system was maintained at 80 °C for an additional 20 min under continuous stirring. This resulted in a homogeneous mixture containing 1% polymer (a 1:1 mass ratio of xanthan to PVA), 0.02 g of CA, and a specific amount of CeO_2_NPs depending on the targeted concentration.

After completion of the reaction, the water bath temperature was reduced to 65 °C, and the solution was held at this temperature for 10 min to equilibrate. The final mixture was then poured into a clean, flat Teflon Petri dish and allowed to dry at room temperature for up to three days in a ventilated hood to ensure slow and uniform solvent evaporation. Once dry, the films were subjected to vacuum drying at room temperature for a minimum of 24 h, followed by storage in a desiccator. To complete the cross-linking process with citric acid, the films were thermally treated at atmospheric pressure in a laboratory oven for seven minutes at 165 °C. The films were then cooled and stored for further characterization. All films were stored in a desiccator until further use to ensure consistent moisture content prior to testing and analysis.

### 4.3. Methods of Film Characterization

#### 4.3.1. FTIR Spectroscopy

FTIR spectroscopy was employed to investigate the chemical structure of the hydrogel films. Measurements were carried out on an IRAffinity-1S spectrophotometer (Shimadzu Corporation, Kyoto, Japan) equipped with an ATR accessory, covering the spectral range of 4000–600 cm^−1^ with a resolution of 4 cm^−1^.

#### 4.3.2. Swelling Behavior

The swelling behavior of the XG/PVA/CeO_2_NPs nanocomposite films was evaluated using a standard gravimetric method [66]. Approximately 10 mg of dried samples were immersed in distilled water at room temperature, and the samples were periodically removed, gently blotted with filter paper to remove surface water, and weighed. All swelling experiments were carried out in triplicate, and the results are reported as mean values ± standard deviation.

#### 4.3.3. DSC

The DSC analyses were conducted using a Maia F3 200 DSC instrument (Netzsch–Gerätebau GmbH, Selb, Germany). About 5 mg of each sample was placed in aluminum crucibles with pierced and tightly sealed lids. Samples were heated at a rate of 10 °C min^−1^ under a nitrogen atmosphere with a flow rate of 50 mL min^−1^. The instrument was calibrated using indium as the standard.

#### 4.3.4. SEM

The cross-sectional morphology of the lyophilized Xn-based nanocomposite films was examined using an environmental scanning electron microscope (ESEM, Quanta 200, FEI) (FEI Company, Hillsboro, OR, USA) operated at 20 kV under low-vacuum conditions. To evaluate the elemental composition of cross-linked films, a SEM, coupled with Energy Dispersive X-ray (EDX) spectroscopy analyzer (Octane Elect Super SDD detector, FEI Company, Hillsboro, OR, USA) was used.

#### 4.3.5. AFM

Atomic force microscopy (AFM) was performed using a NTEGRA scanning probe microscope (NT-MDT Spectrum Instruments, Moscow, Russia) to obtain 2D and 3D surface topography of the Xn-based nanocomposite films. The scans were carried out with rectangular silicon cantilevers (NSG 03, NT-MDT Spectrum Instruments, Moscow, Russia) featuring uniform tip height and aspect ratio. Image processing and surface parameter analysis were conducted with Nova software (v.19891, Solver) [67].

#### 4.3.6. UV–Vis Spectroscopy

Ultraviolet–visible (UV–Vis) transmission spectra were recorded using a Shimadzu U-4100 spectrophotometer. Diffuse reflectance spectra were obtained with the same instrument equipped with an integrating sphere accessory.

### 4.4. Sorption Experiments

The adsorption of MB and CV onto Xn-based hydrogel films was investigated using a batch method. Experiments were conducted at 25 °C by immersing 0.05 g of dried hydrogel samples into 10 mL of dye solutions with initial concentrations starting from 50 mg L^−1^. All solutions were prepared in Milli-Q water at pH 5.8. The vials containing dye solutions and hydrogel films were placed on a multi-position magnetic stirrer and agitated at ~250 rpm for 24 h. Following this period, the films were separated by filtration, and the residual dye concentration in the filtrate was quantified by UV–Vis spectroscopy (SPECORD 200, Carl Zeiss, Jena, Germany) at 590 nm for CV and 664 nm for MB. The decrease in absorbance at the characteristic wavelengths was used to determine the dye uptake.

The equilibrium adsorption capacity (q_e_, mg g^−1^) was calculated using Equation (2) [68,69]:

(2) $$q_{e}=\frac{\left(C_{0}-C_{e}\right)V}{W}$$

where C_0_ and C_e_ (mg L^−1^) are the initial and equilibrium dye concentrations, respectively, V (L) is the solution volume, and W (g) is the mass of the hydrogel adsorbent. The average of three independent replicates was reported for each sorption experiment.

The removal efficiency was calculated with Equation (3) [70]:

(3) $$RE\%=\frac{\left(C_{0}-C_{e}\right)}{C_{0}}$$

where C_0_ and C_e_ have the same meaning as in Equation (2).

For each adsorption experiment, the average of three replicates was reported.

### 4.5. Desorption of Dyes

Dye desorption from the nanocomposite films was performed through a two-step chemical regeneration process, according to a protocol already established for chitosan-based nanocomposite hydrogels [69]. In the first step, the films were exposed to 0.1 M HCl (20 mL, 2 h) followed by immersion in 50% methanol (10 mL, ~20 h). Afterward, they were rinsed with distilled water until near-neutral pH was obtained. The second step involved five successive treatments with 0.1 M NaOH (50 mL, 2.5 h each), followed by thorough washing with distilled water to restore neutrality.

## Figures and Tables

**Table 1:** Samples code and their composition.

^1^ Sample Code	Xn/PVA % Weight Ratio	% CeO_2_ NPs	Thermal Treatment, °C
P0	50/50	0	-
P0-T	50/50	0	165
P5	50/50	5	-
P5-T	50/50	5	165
P10	50/50	10	-
P10-T	50/50	10	165
P15	50/50	15	-
P15-T	50/50	15	165

^1^ P0—blank polymer matrix; P0-T—blank thermal treated sample.

**Table 2:** Root Mean Square in nm of nanocomposite films obtained from AFM images.

Samples	5 × 5 µm^2^	10 × 10 µm^2^	20 × 20 µm^2^
P0	1.473	3.267	9.401
P10	1.822	3.961	24.367
P10-T	2.142	-	31.987

**Table 3:** Optical parameters for CeO_2_NPs and doped polymeric films obtained by Tauc and Urbac representation.

Samples	$E_{g}$ (eV)				$E_{U}$ (meV)	${∆E}_{U}$ (%)
	*n* = 1/2		*n* = 2			
CeO_2_	3.173	4.102	2.951	4.042	282.75	-
P5	3.18	4.043	3	4.009	259.39	-
P10	3.182	4.058	2.977	4.051	223.65	-
P15	3.153		2.912		213.90	-
P5-T	3.110		3.023		505.43	94.85
P10-T	3.120		3.014		345.03	54.27
P15-T	3.121		3.006		326.51	52.65

**Table 4:** Comparison of the removal efficiency (RE) of different reported sorbents with the present Xn/PVA/CeO_2_ nanocomposite hydrogel films for MB and CV removal.

^1^ Sorbent	Dye Type	Sorbent Dose, g/L	pH	RE%	Reference
TiO_2_-embedded guar gum hydrogel	MB	-	10	~93%	[8]
Gum ghatti-*g*-PAMPS/Fe_3_O_4_ hydrogel nanocomposite	MB	10	2–10	~63.5–78%	[9]
CMC-*g*-PAAm/CL-Fe_3_O_4_	MB	1.5	9	~95.01%	[10]
HPAM/SiO_2_@Xn	CV	20	7	~95%	[12]
TiO_2_-containing Gum ghatti-*cl*-PAAm composite hydrogel	MB	-	7	~98%	[18]
Cross-linked Acrylate Xn	MB	1.2	11	>95%	[39]
Xn-*cl*-2-(N-morpholinoethyl methacrylate)/TiO_2_ hydrogels	MB	40	11	~80%	[41]
Xn-*cl*-2-(N-morpholinoethyl methacrylate)/TiO_2_ hydrogels	CV	40	11	~60%	[41]
Xn/PVA/CeO_2_ nanocomposite hydrogel films	MB	5	5.8	~97–98%	This study
Xn/PVA/CeO_2_ nanocomposite hydrogel films	CV	5	5.8	~71–83%	This study

^1^ PAMPS—poly(2-acrylamido-2-methylpropane sulfonic acid); CMC—carboxymethyl cellulose; PAAm—poly (acrylamide); CL—biochar of *Luffa Cylindrica*; HPAM—hydrolyzed polyacrylamide; *cl*—cross-linked.

## Data Availability

The original contributions presented in this study are included in the article/Supplementary Materials. Further inquiries can be directed to the corresponding author.

## Supplementary Materials

The following supporting information can be downloaded at: https://www.mdpi.com/article/10.3390/gels11100809/s1. Figure S1: XRD diffractogram of CeO_2_NPs; Figure S2: TEM images of CeO_2_NPs; Figure S3: Streaming potential measurements on CeO_2_NPs as a function of pH; Figure S4: ATR-FTIR spectra of Xn/PVA/CeO_2_ nanocomposite films containing 5% CeO_2_NPs before and after thermal treatment; Figure S5: ATR-FTIR spectra of blank polymer matrix without nanoparticles before and after thermal treatment and of Xn/PVA/CeO_2_ nanocomposite films containing 10% CeO_2_NPs before and after thermal treatment; Figure S6: ATR-FTIR spectra of Xn/PVA/CeO_2_ nanocomposite films containing 15% CeO_2_NPs before and after thermal treatment; Figure S7: EDX profiles of the cross-linked nanocomposite films; Table S1: The percentage of each element on the surface of the cross-linked nanocomposite films; Figure S8: Reflectance spectra (a) and transmittance (b) for polymer matrix (P0) and thermal aging at 165 °C of polymer matrix (P0-T), without nanoparticles content; Figure S9: Reflectance spectra (a,c) and transmittance (b,d) for polymer films doped with CeO_2_ with content of 5, 10 and 15% for untreated, P5, P10, P15 and 165 °C thermal treated, P5-T, P10-T, P15-T, composites; Table S2: The values of the optical parameters obtained from the first derivative of the spectra and the Tauc equation; Figure S10: The representation for calculating *n*, using the estimated value of Eg from the derivative of Tauc equation; Figure S11: The Tauc representation of CeO_2_NPs for the direct (a) and indirect (b) optical transition; Figure S12: Tauc representation for the doped non-cross-linked polymeric films, P5 (a,b), P10 (c,d), and P15 (e,f) for direct (*n* = 1/2) and indirect (*n* = 2) allowed optical transitions; Figure S13: Tauc representation for the doped cross-linked polymeric films, P5-T (a,b), P10-T (c,d), and P15-T (e,f) for direct (*n* = 1/2) and indirect (*n* = 2) allowed optical transitions; Figure S14: Optical pictures of nanocomposite films after two-step chemical regeneration strategy that combined acidic/organic and alkaline treatments. (A) P0-T films after removal of MB; (B) P0-T films after removal of CV; (C) P10-T films after removal of MB; (D) P10-T films after removal of CV. References [47,48,49,50,51,52,53,54,55,56,57,58,59,60,63,64,66] are cited in the Supplementary Materials.
