# Supplementary material for: Preparation and Characterization of Chemically Cross-Linked Xanthan/Poly(Vinylalcohol) Hydrogel Films Containing Cerium Oxide Nanoparticles for Potential Application in Removal of Methylene Blue and Crystal Violet Dyes
Source: Gels. 2025 Oct 9;11(10):809. doi: 10.3390/gels11100809 (PMC12564716; doi:10.3390/gels11100809)
Supplement: Supplementary file 1 [file gels-11-00809-s001.zip › gels-3893409-supplementary.pdf]

Supplementary Materials

Preparation and Characterization of Chemically Cross-Linked Xanthan/Poly(Vinylalcohol) Hydrogel Films Containing Cerium Oxide Nanoparticles for Potential Application in Removal of Methylene Blue and Crystal Violet Dyes

Nicusor Fifere *, Maria Marinela Lazar, Irina Elena Raschip, Anton Airinei, Cristian-Dragos Varganici and Maria Valentina Dinu

"Petru Poni" Institute of Macromolecular Chemistry, Grigore Ghica Voda Alley 41A, 700487 Iasi, Romania

* Correspondence: fifere.nicusor@icmpp.ro

Figure S1. XRD diffractogram of CeO_2NPs
Figure S2. TEM images of CeO_2NPs
Figure S3. Streaming potential measurements on CeO_2NPs as a function of pH.
Figure S4. ATR-FTIR spectra of Xn/PVA/CeO_2 nanocomposite films containing 5% CeO2NPs before and after thermal treatment.
Figure S5. ATR-FTIR spectra of blank polymer matrix without nanoparticles before and after thermal treatment and of Xn/PVA/CeO_2 nanocomposite films containing 10% CeO_2NPs before and after thermal treatment.
Figure S6. ATR-FTIR spectra of Xn/PVA/CeO_2 nanocomposite films containing 15% CeO2NPs before and after thermal treatment.
Figure S7. EDX profiles of the cross-linked nanocomposite films.
Table S1. The percentage of each element on the surface of the cross-linked nanocomposite films.
Figure S8. Reflectance spectra (a) and transmittance (b) for polymer matrix (P0) and thermal aging at 165 ^{o}C of polymer matrix (P0-T), without nanoparticles content.

Figure S9. Reflectance spectra (a and c) and transmittance (b and d) for polymer films doped with CeO_2 with content of 5, 10 and 15% for untreated, P5, P10, P15 and 165° C thermal treated, P5-T, P10-T, P15-T, composites.
Table S2. The values of the optical parameters obtained from the first derivative of the spectra and the Tauc equation.
Figure S10. The representation for calculating n, using the estimated value of Eg from the derivative of Tauc equation.
Figure S11. The Tauc representation of CeO_2NPs for the direct (a) and indirect (b) optical transition.
Figure S12. Tauc representation for the doped non-cross-linked polymeric films, P5 (a and b), P10 (c and d), and P15 (e and f) for direct (n = 1/2) and indirect (n = 2) allowed optical transitions.
Figure S13. Tauc representation for the doped cross-linked polymeric films, P5-T (a and b), P10-T (c and d), and P15-T (e and f) for direct (n = 1/2) and indirect (n = 2) allowed optical transitions.

Figure S14. Optical pictures of nanocomposite films after two-step chemical regeneration strategy that combined acidic/organic and alkaline treatments. (A) P0-T films after removal of MB; (B) P0-T films after removal of CV; (C) P10-T films after removal of MB; (D) P10-T films after removal of CV.

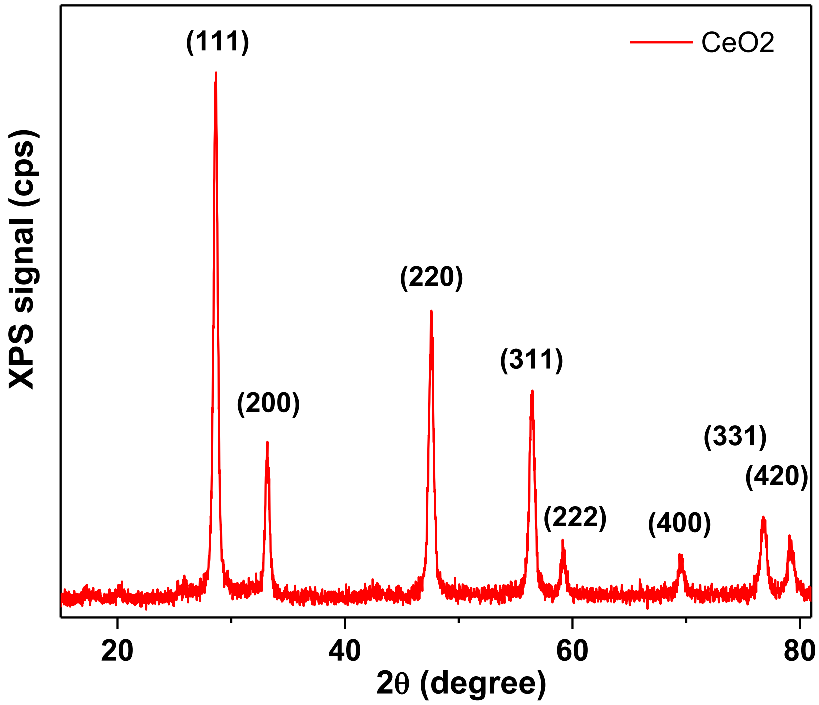

Figure S1. XRD digractogram of CeO_2NPs

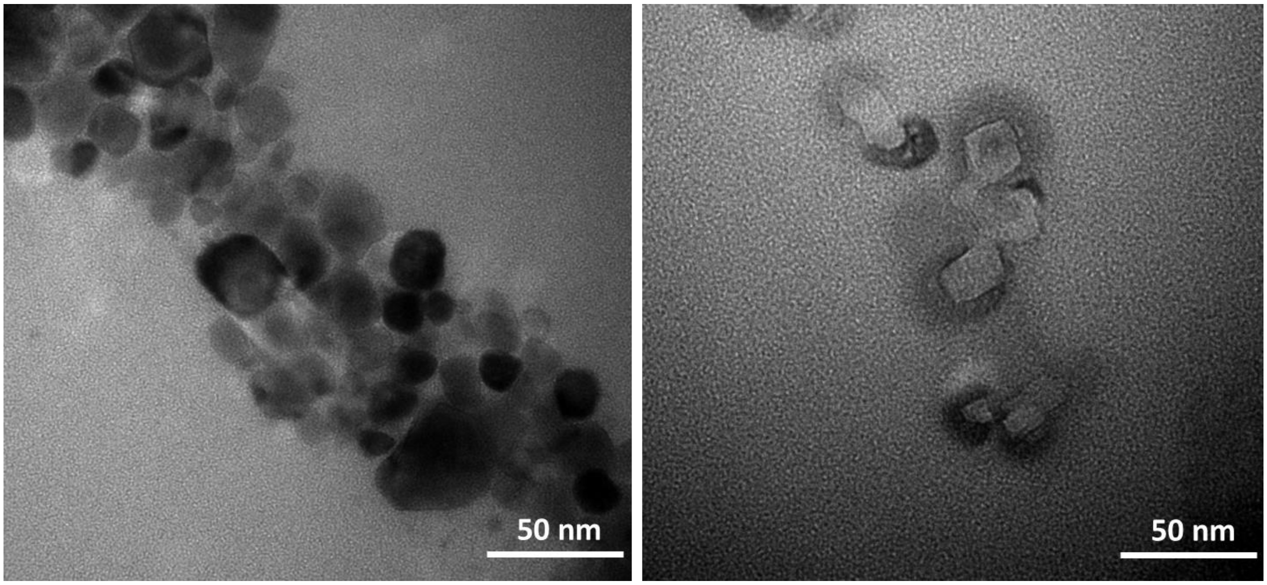

Figure S2. TEM images of CeO_2NPs

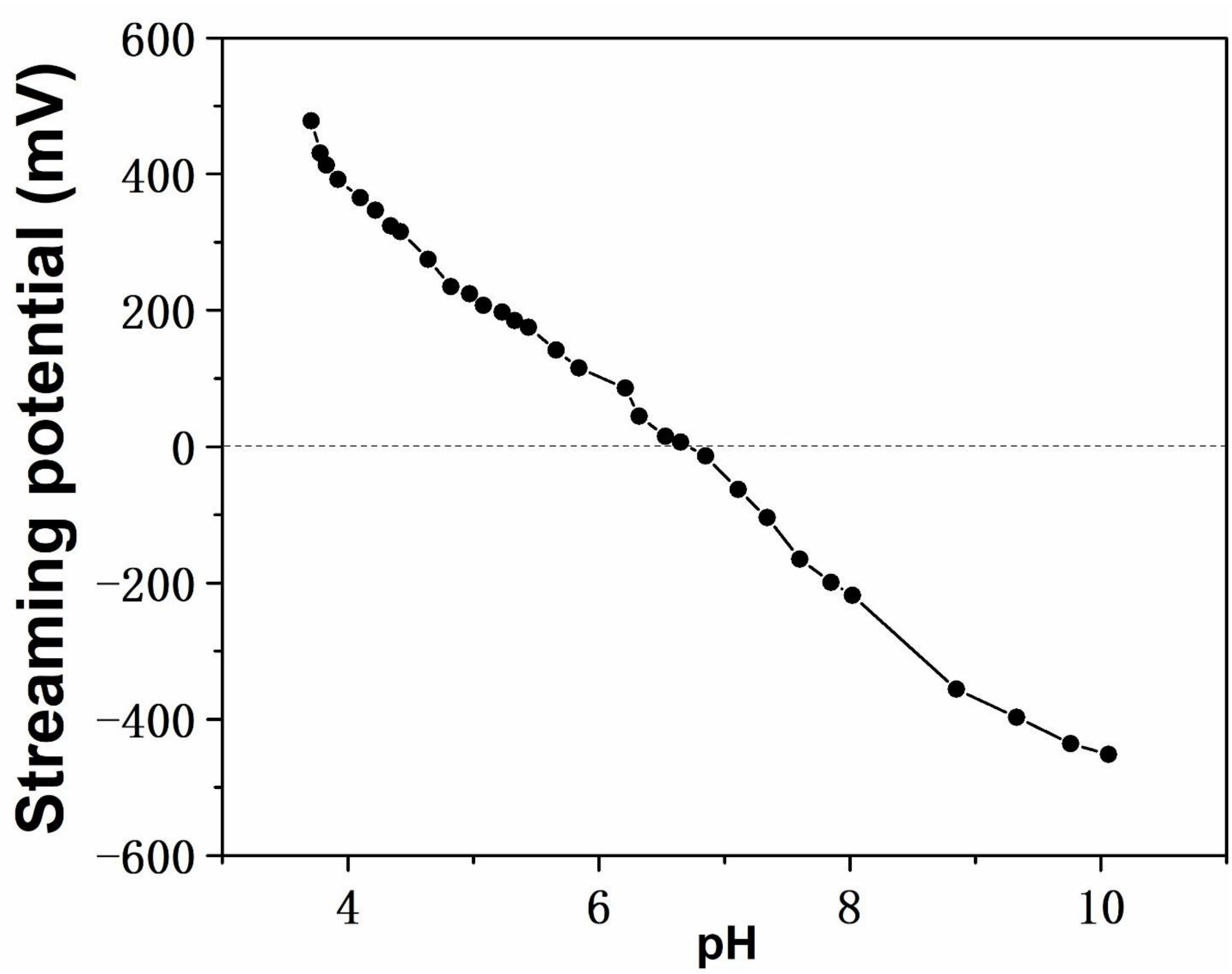

Figure S3. Streaming potential measurements on CeO_2NPs as a function of pH. Surface charge at the pH used for films preparation (~5.8) is slighty positive for CeO_2NPs.

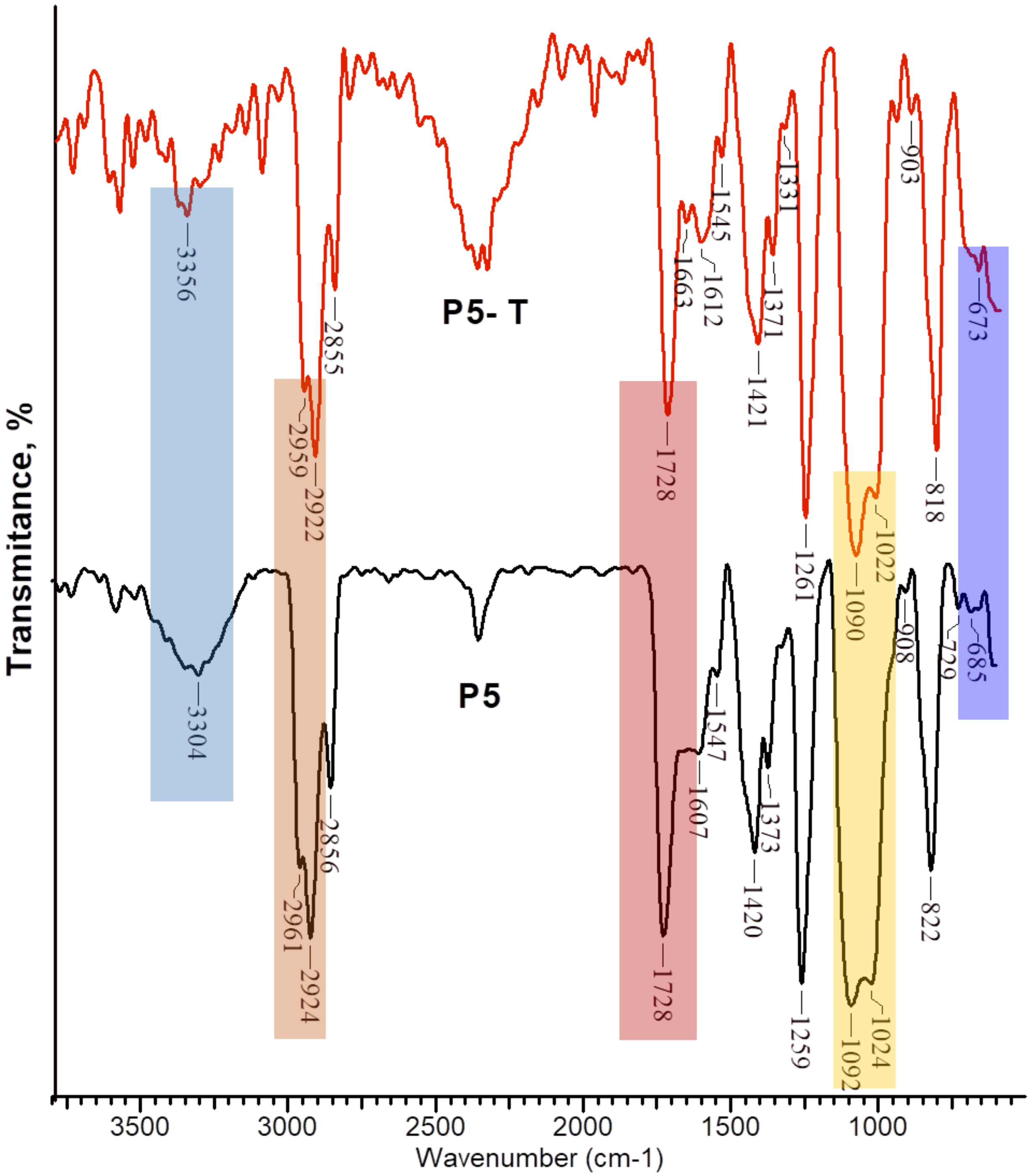

Figure S4. ATR-FTIR spectra of Xn/PVA/CeO_2 nanocomposite films containing 5% CeO_2NPs before and after termal treatment.

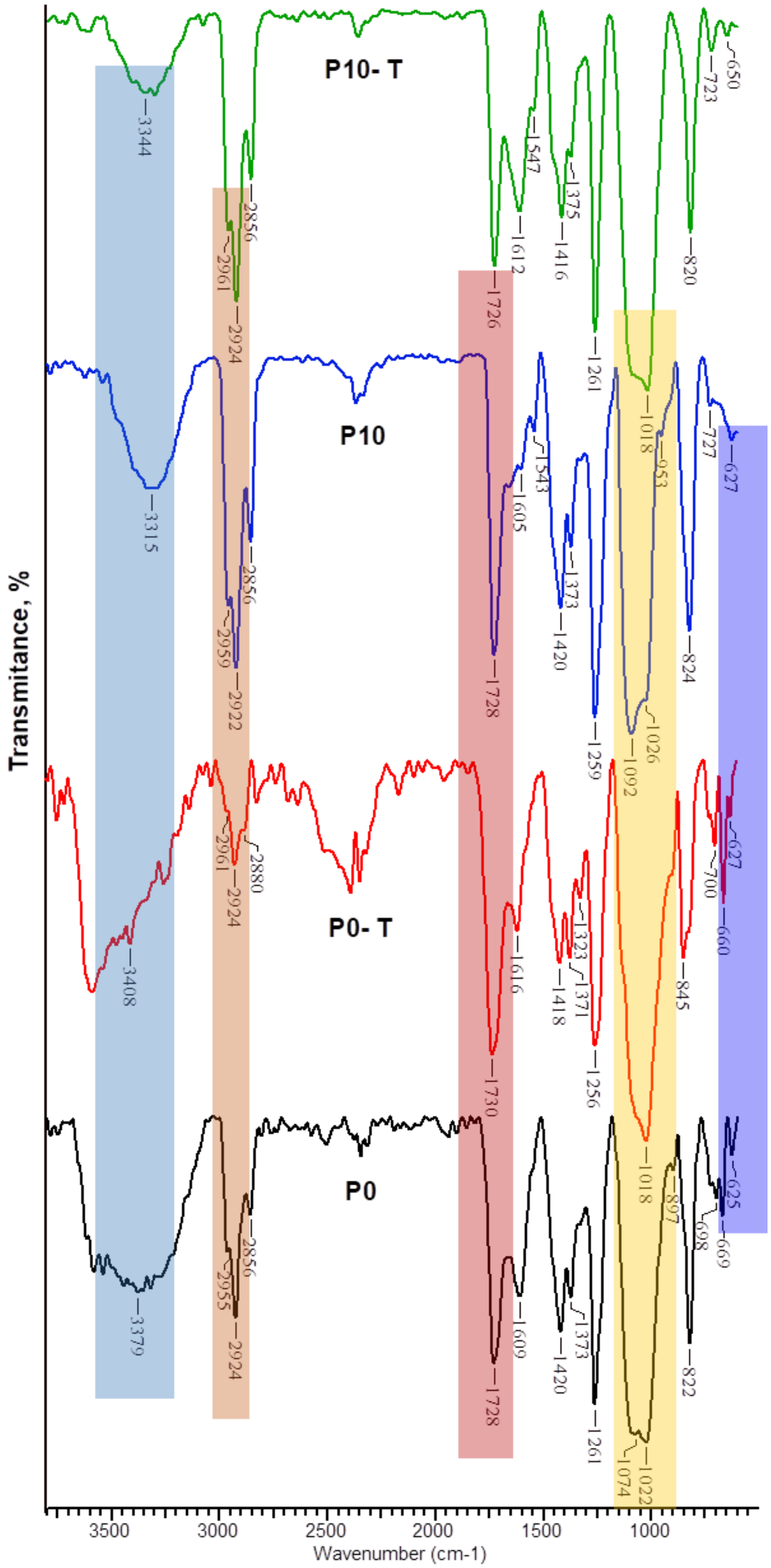

Figure S5. ATR-FTIR spectra of blank polymer matrix without nanoparticles before and after thermal treatment and of Xn/PVA/CeO_2 nanocomposite films containing 10% CeO_2NPs before and after thermal treatment.

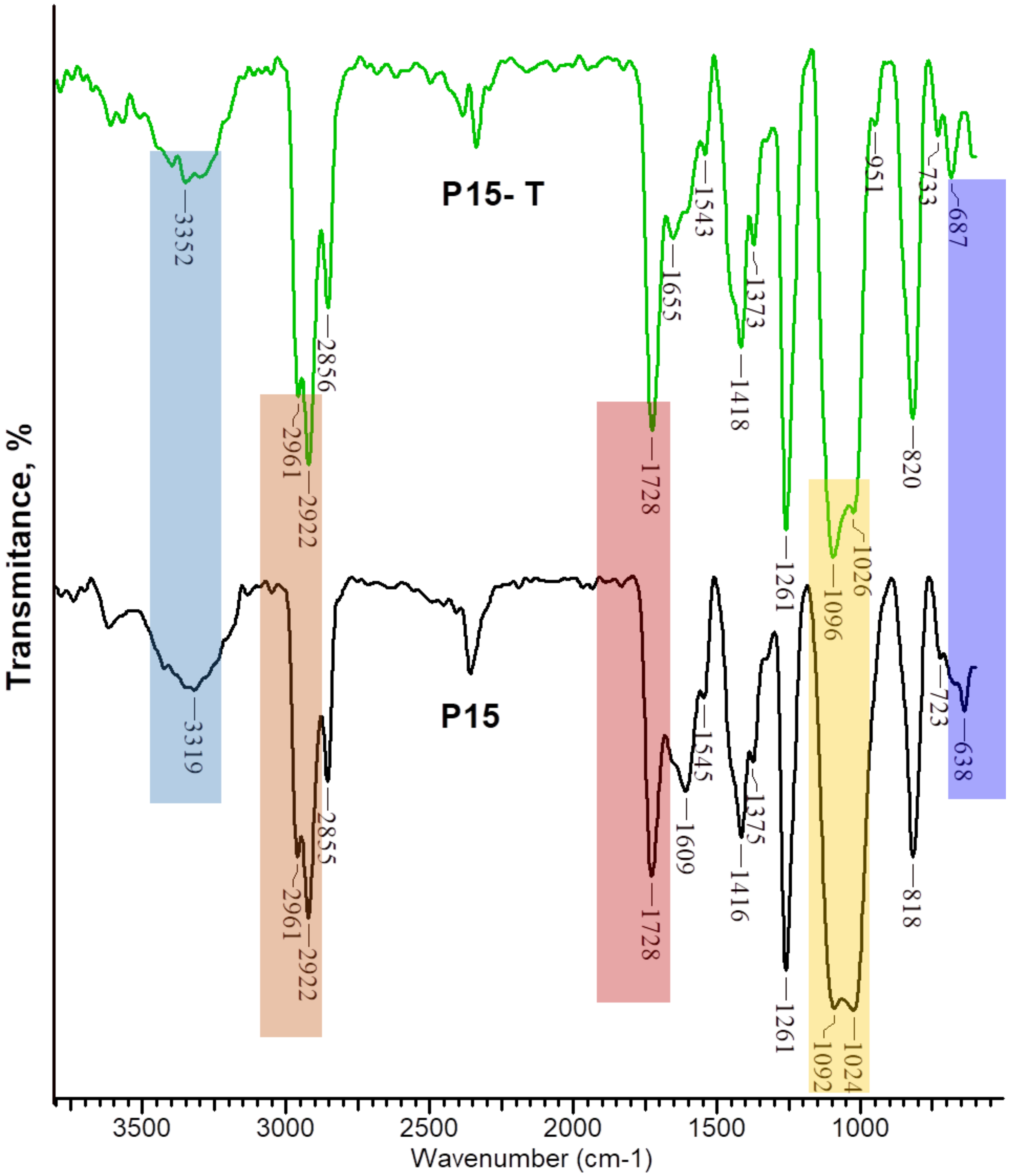

Figure S6. ATR-FTIR spectra of Xn/PVA/CeO_2 nanocomposite films containing 15% CeO_2NPs before and after termal treatment.

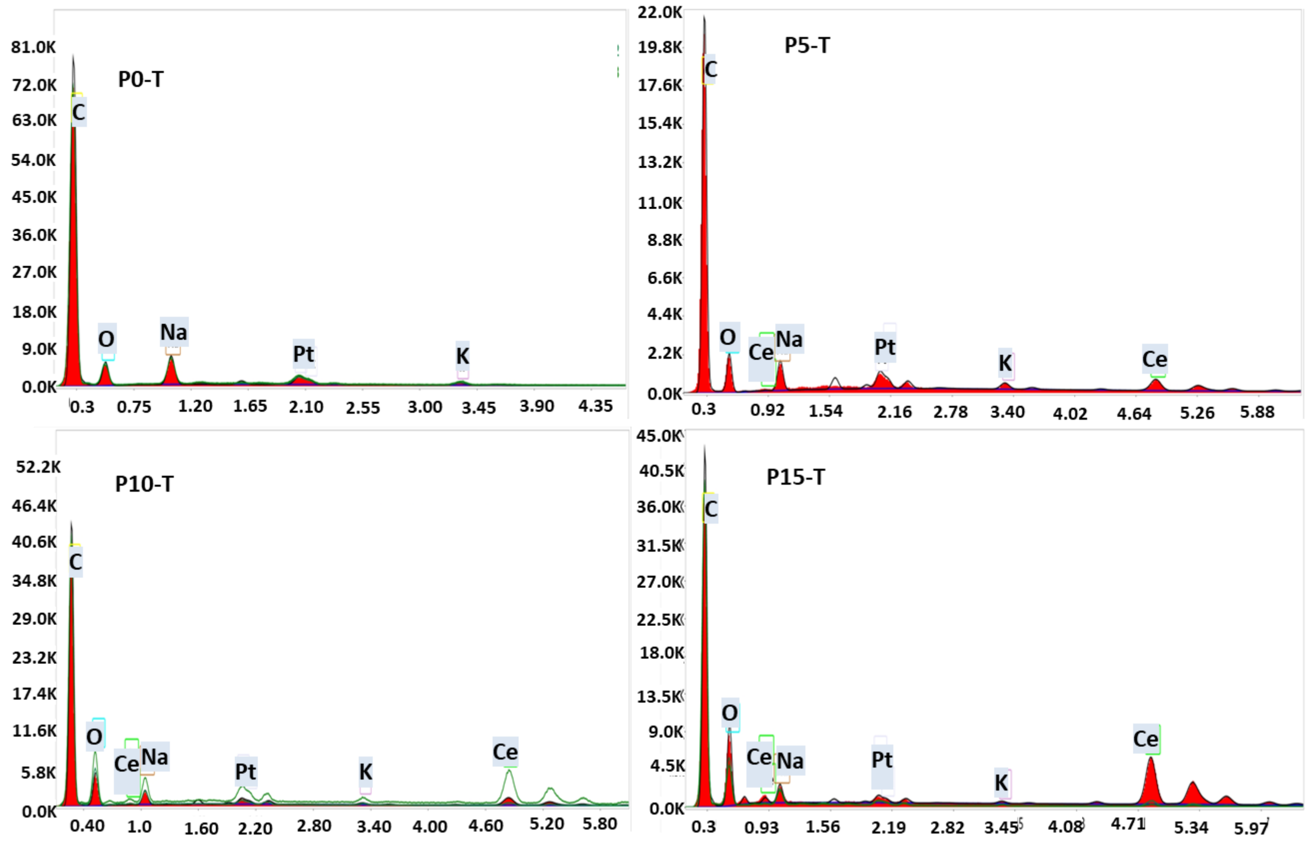

Figure S7. EDX profiles and the percentage of each element on the surface of the cross-linked nanocomposite films.

Table S1. The percentage of each element on the surface of the cross-linked nanocomposite films.

Sample/ Element	P0-T		P5-T		P10-T		P15-T	
	Wt%	At%	Wt%	At%	Wt%	At%	Wt%	At%
C	76.3	84.5	67.4	81.8	50.7	72.8	50.2	72.2
O	14.3	11.9	15.3	13.9	18.9	20.3	20.6	22.3
Na	5.5	3.2	4.6	2.9	5	3.7	3.1	2.3
K	0.6	0.2	0.9	0.3	0.6	0.3	0.3	0.1
Ce	-	-	6.9	0.7	19.5	2.4	24.1	3
Pt	3.3	0.2	5	0.4	5.3	0.5	1.7	0.2

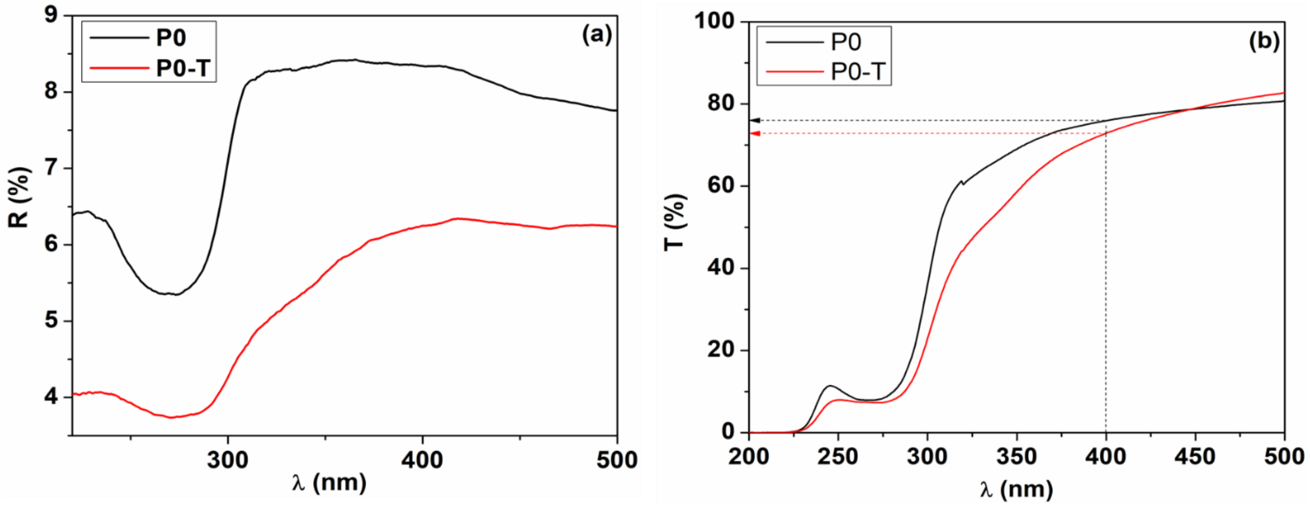

Figure S8. Reflectance spectra (a) and transmittance (b) for polymer matrix (P0) and thermal aging at 165 ^{o}C of polymer matrix (P0-T), without nanoparticles content.

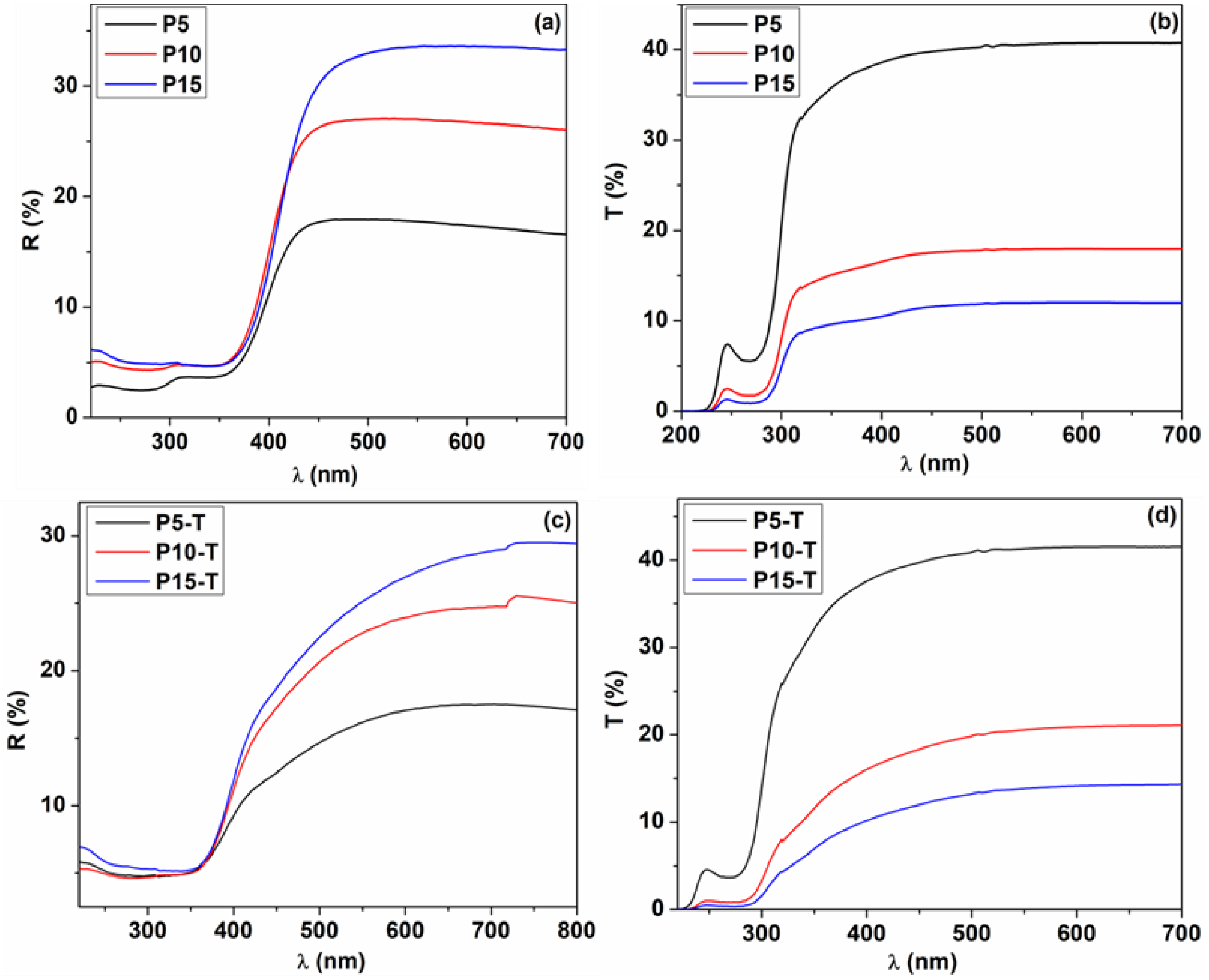

Figure S9. Reflectance spectra (a and c) and transmittance (b and d) for polymer films doped with CeO_2 with content of 5, 10 and 15% for untreated, P5, P10, P15 and 165° C thermal treated, P5-T, P10-T, P15-T, composites.

Table S2. The values of the optical parameters obtained from the first derivative of the spectra and the Tauc equation.

Sample	Wavelength position (nm) of first derivative maximum of the spectrum		Values estimate from the first derivation of Tauc equation	
	T%	R%		
	λ (nm)	λ (nm)	E_{max} (eV)	n
P0	302	299	4.147	0.15
P0-T	302	301	4.119	0.1
P5	300	399	3.179	0.5
P5-T	299	399	3.179	0.32
P10	299	400	3.179	0.51
P10-T	302	398	3.179	0.4
P15	301	402	3.092	0.63
P15-T	302	398	3.171	0.41
CeO_2NPs	-	401	3.179	0.51

Bandgap determination

To determine the type of electronic transition and the value of the optical bandgap, diffuse reflectance spectra were analyzed using the modified Tauc equation in combination with the Kubelka–Munk function [1]:

$$[F(R_\infty)E]^{1/n} = B(E - E_g) \quad \text{(S1)}$$

Where, B is a constant, $E = h\nu$ the energy and E_g represent the gap energy, $F(R_\infty)$ is the Kubelka–Munk function, which substitutes the absorption coefficient in the Tauc equation and is defined as:

$$F(R_\infty) = (1 - R_\infty^2)/(2R_\infty) \quad \text{(S2)}$$

with $R_\infty = R_{sample}/R_{standard}$

The type of electronic transition is determined by the exponent n, which takes the values 1/2 for a direct allowed transition and 2 for an indirect allowed transition. It should be emphasized that Equation (S1) is valid under the assumption of perfectly diffuse scattering (at a 60° incidence angle), where $F(R_\infty)$ is proportional to the linear absorption coefficient.

When n is known, the optical bandgap can be obtained from the linear extrapolation of $[F(R_\infty)E]^{1/n}$ vs. E to $F(R_\infty) = 0$, corresponding to the linearized form of Equation (S1) [2-4]:

$$[F(R_\infty)E]^{1/n} = -BE_g + BE \quad \text{(S1.a)}$$

CeO_2NPs may exhibit both direct and indirect transitions. To identify the transition type, the logarithmic form of Equation (S3) was employed:

$$ln(F \cdot E) = n \cdot lnB + n \cdot \ln(E - E_{est}) \quad \text{(S3)}$$

where E_{est} – is the energy estimated from the asymptote obtained from representation of derivative of $ln(F \cdot E)$ vs. energy [5] as can be seen from equation obtained by derivation of Equation (S3) [6]:

$$\frac{dln(F \cdot E)}{dE} = \frac{n}{E - E_g} \quad \text{(S4)}$$

In Equation (S4), for E close to E_g, there appears an asymptotic dependence of the derivative as a function of energy, which shows an asymptotic dependence on energy, which experimentally manifests as a maximum in the derivative plot. The position of this asymptote provides the estimated bandgap value, $E_{g,est}$. Using this value, the exponent n can be determined from the slope of the linearized representation of Equation (S3).

Analysis of the derivative form of the Tauc relation (Figure S10), applied to CeO_2NPs deposited on quartz glass, clearly indicates n = 0.5, confirming a direct allowed transition. In contrast, the values of n obtained for the polymer film without nanoparticles (sample P0) are inconclusive, as summarized in Table 3. However, for all samples containing nanoparticles, the slopes converge toward n = 0.5, indicating that the direct optical transition is predominant. It should be noted that the experimental data may be influenced by the surrounding polymer matrix. In this context, the

slight deviations of n from 0.5 observed in polymer–nanoparticle films are likely due to the matrix contribution. For this reason, the linearity of Equation (S1a, Supporting Information) was evaluated for both direct and indirect transitions in all samples, and the corresponding bandgap energies were determined. The Tauc representation of the reflectance spectra, expressed as $[F(R_\infty)E]^{1/n} = f(E)$, for CeO_2NPs is shown in Figure S11. Two linear regions can be distinguished in both the direct and indirect representations, with the corresponding bandgap energies summarized in Table 4. These two linear regions arise because the Tauc representation is strongly affected by the density of defect states within the bandgap. The extracted gap energies are consistent with values reported in the literature [7-10] and generally correspond either to transitions between the valence band and the extended 4f orbital within the forbidden gap (originating from cerium and often considered as part of the conduction band) [11,12] or to transitions involving isolated states localized below the extended 4f orbital.

Theoretical calculations predict a direct interband transition at approximately 4 eV, with the extended 4f orbital as the final state [13,14]. Accordingly, the gap energies slightly above 4 eV obtained in our experiments (Table 4) can be attributed to interband optical transitions from the valence band to the 4f conduction band. These values were observed for both bare CeO_2NPs and the nanocomposite hydrogel films.

In addition, the presence of localized states within the forbidden region, close to the conduction band, can give rise to sub-band optical transitions, resulting in apparent bandgap values below 4 eV, as reflected in Table 4. Furthermore, the bandgap energies corresponding to indirect transitions are slightly lower than those of direct transitions, due to the involvement of phonons in the former [15].

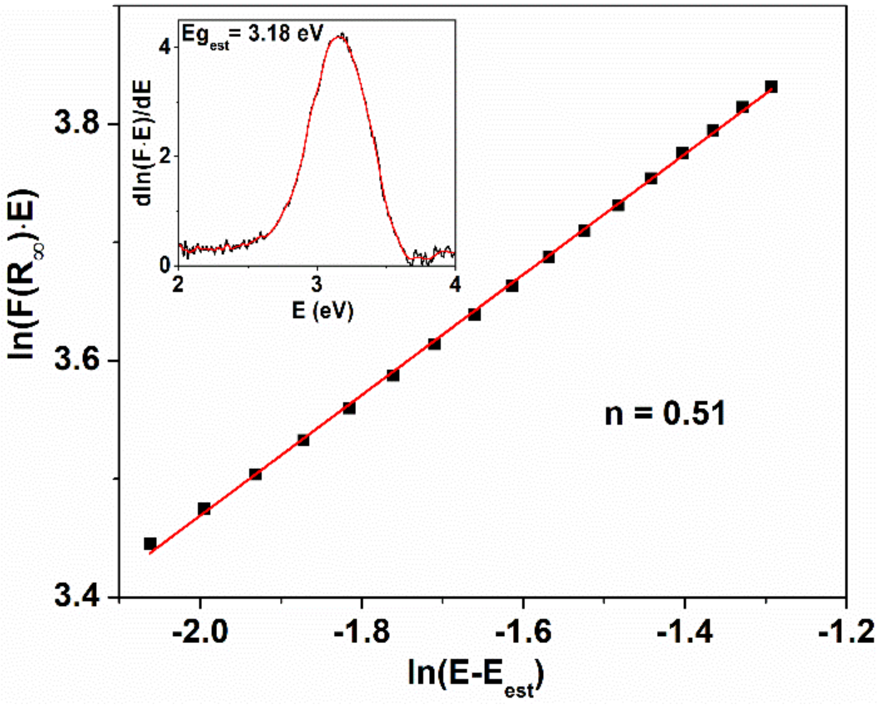

Figure S10. The representation for calculating n, using the estimated value of Eg from the derivative of Tauc equation.

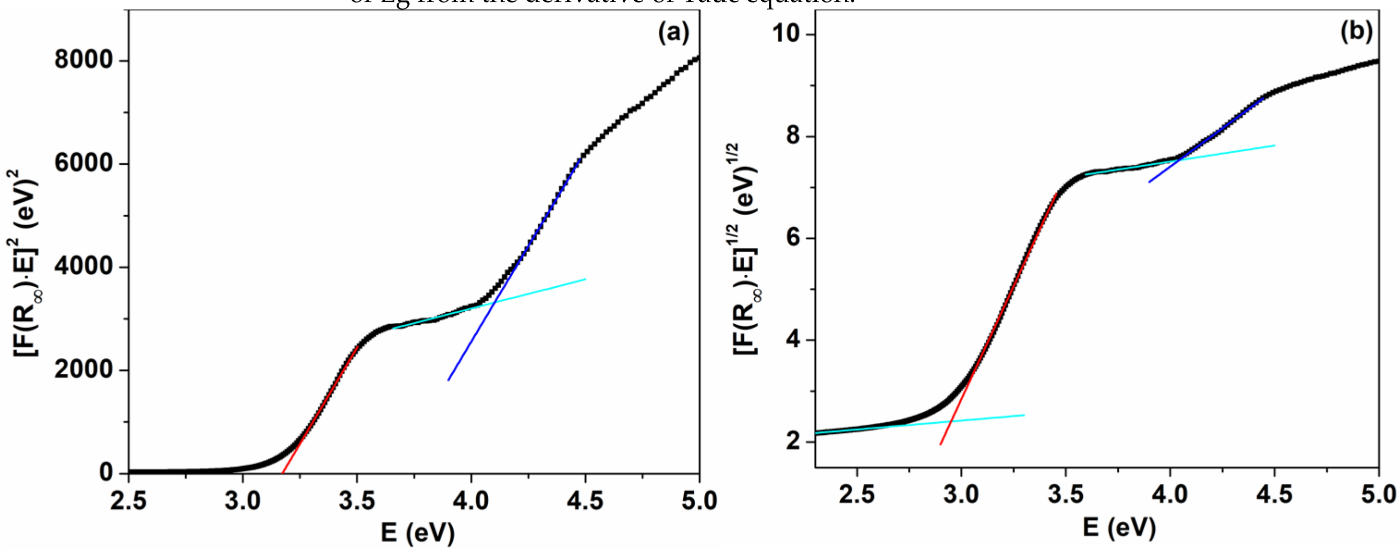

Figure S11. The Tauc representation of CeO_2NPs for the direct (a) and indirect (b) optical transition.

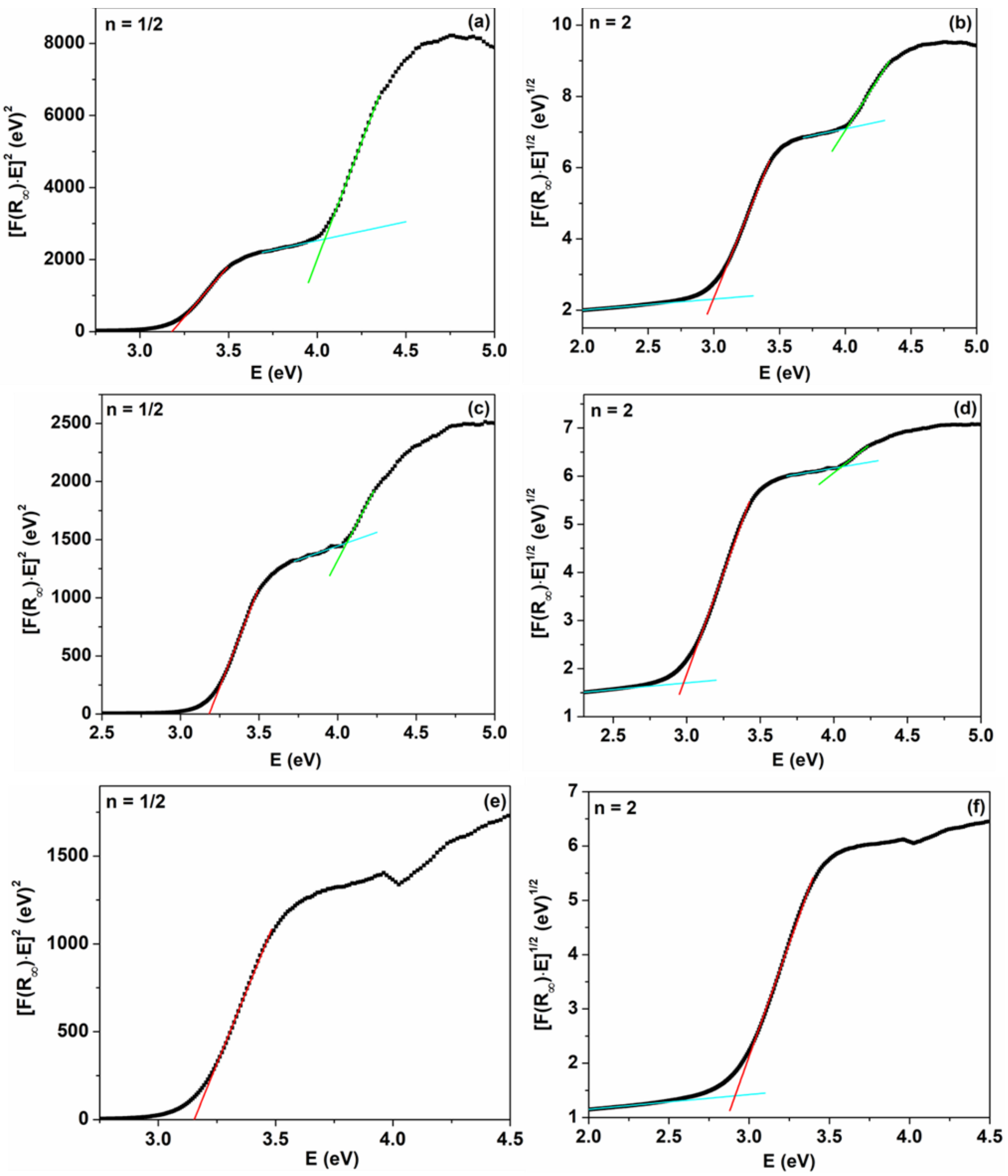

Figure S12. Tauc representation for the doped non-cross-linked polymeric films, P5 (a and b), P10 (c and d), and P15 (e and f) for direct (n = 1/2) and indirect (n = 2) allowed optical transitions.

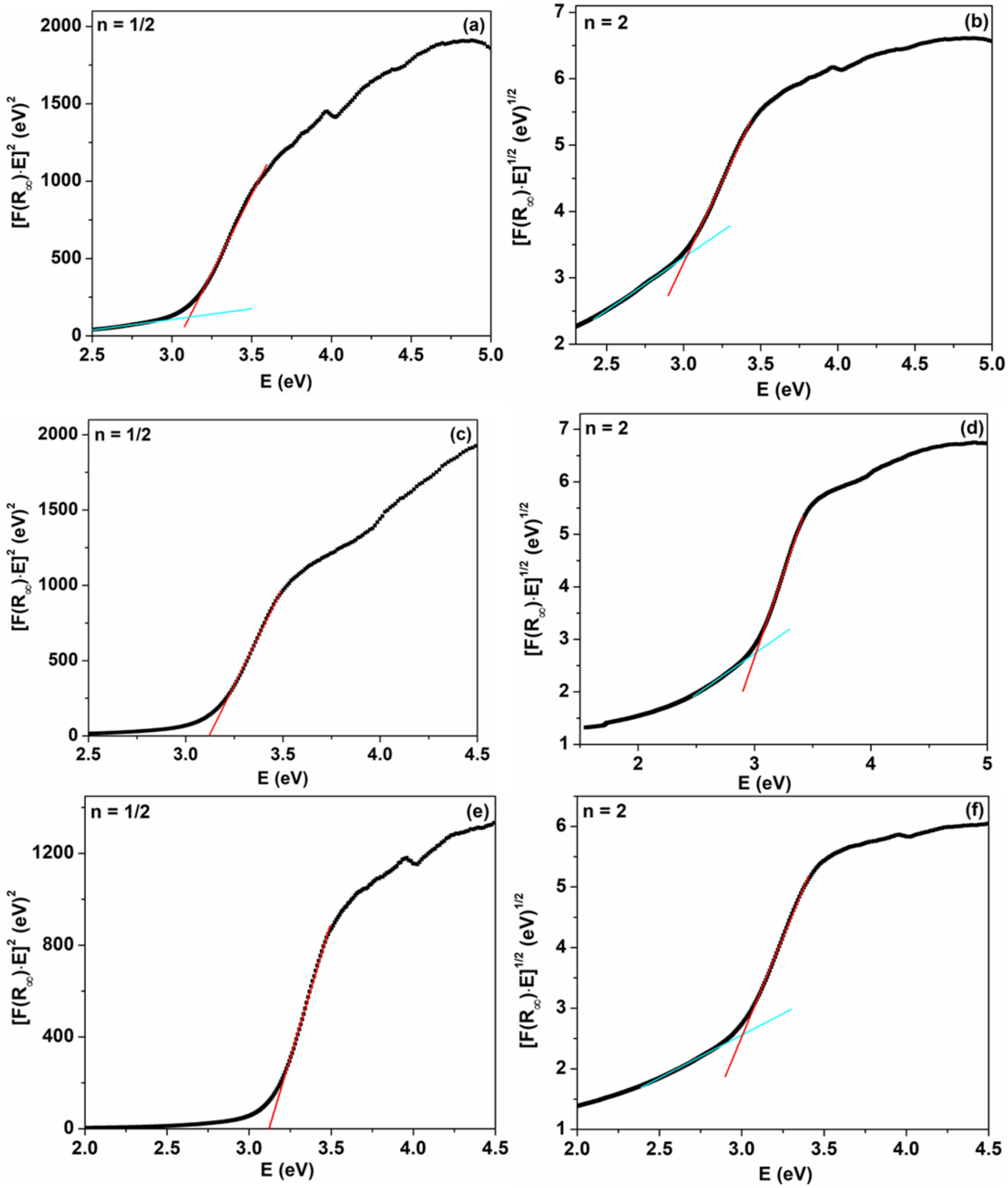

Figure S13. Tauc representation for the doped cross-linked polymeric films, P5-T (a and b), P10-T (c and d), and P15-T (e and f) for direct (n = 1/2) and indirect (n = 2) allowed optical transitions.

Urbach energy analysis

The evaluation of localized states within the forbidden band is based on the empirical law describing the exponential dependence of the absorption coefficient on photon energy for transitions occurring below and near the band edge. This region is commonly referred to as the Urbach tail

[16]. By incorporating the Kubelka–Munk function, the exponential relation can be reformulated as [17]:

$$lnF(R_\infty) = ln\beta + \frac{E}{E_U} \quad \text{(S5)}$$

where β is a constant and E_U represent the Urbach energy, which reflects the width of the band tail localized in the bandgap near the absorption edge.

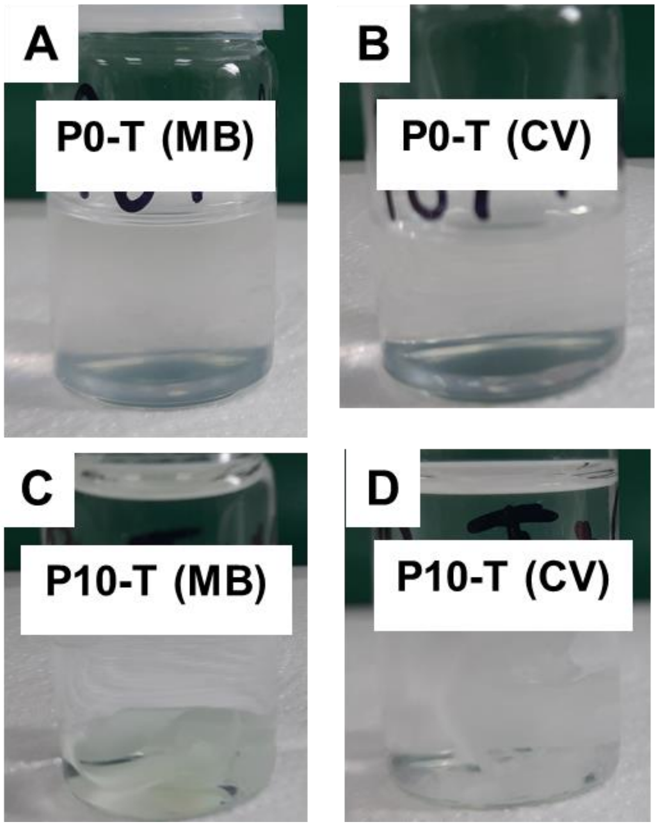

Figure S14. Optical pictures of nanocomposite films after two-step chemical regeneration strategy that combined acidic/organic and alkaline treatments. (A) P0-T films after removal of MB; (B) P0-T films after removal of CV; (C) P10-T films after removal of MB; (D) P10-T films after removal of CV.

As Figure S14 shows, only the chemically cross-linked films containing CeO_2NPs were stable after these treatments and can be reused in another sorption cycle.

Swelling behavior evalution

From these measurements, the equilibrium water content (EWC) and the swelling ratio (SR) were calculated using Equations (S6) and (S7), respectively [18].

$$SR = \frac{W_t}{W_d} \quad \text{(S6)}$$

$$EWC(\%) = \frac{W_{eq} - W_d}{W_{eq}} \times 100 \quad \text{(S7)}$$

W_t denotes the mass of the nanocomposite films at time t, W_d is the mass of the dry films, and W_{eq} represents the mass of the films at swelling equilibrium.

References

1. Romasanta, L.J.; D'Alençon, L.; Kirchner S.; Pradère, C.; Leng, J. Thin coatings of cerium oxide nanoparticles with anti-reflective properties. *Appl. Sci*. **2019**, *9*, 3886.
2. Makuła, P.; Pacia, M.; Macyk, W. How to correctly determine the band gap energy of modified semiconductor photocatalysts based on UV−Vis spectra. *J. Phys. Chem. Lett*. **2018**, *9*, 6814−6817.
3. Morales, A.E.; Mora, E.S.; Pal, U. Use of diffuse reflectance spectroscopy for optical characterization of un-supported nanostructures. *Rev. Mex. Fís.* **2007**, *53*, 18–22.
4. Harynski, Ł.; Olejnik, A.; Grochowska, K.; Siuzdak, K. A facile method for Tauc exponent and corresponding electronic transitions determination in semiconductors directly from UV−Vis spectroscopy data. *Opt. Mater.* **2022,** 127, 112205.
5. Morales, A.E.; Mora, E.S.; Pal, U. Use of diffuse reflectance spectroscopy for optical characterization of un-supported nanostructures. *Rev. Mex. Fís.* **2007**, *53*, 18–22.
6. Łukasz Harynski, Adrian Olejnik, Katarzyna Grochowska, Katarzyna SiuzdakA facile method for Tauc exponent and corresponding electronic transitions determination in semiconductors directly from UV−Vis spectroscopy data. *Optical Materials* **2022,** 127, 112205.
7. Sharma, J.; Singh, G.; Thakur, A.; Saini, G.S.S.; Goyal, N.; Tripathi, S.K. Preparation and characterization of SnSe nanocrystalline thin films. *J. Optoelectron. Adv. Mater*. **2005**, *7*, 2085 – 2094.
8. Calvache-Muñoz, J.; Prado, F.A.; Rodríguez-Páez, J.E. Cerium oxide nanoparticles: Synthesis, characterization and tentative mechanism of particle formation. *Coll. Surf. A*, **2017**, *529*, 146–159.
9. Nadjia, L.; Abdelkader, E.; Naceur, B.; Ahmed, B. CeO_2 nanoscale particles: Synthesis, characterization and photocatalytic activity under UVA light irradiation. *J. Rare Earth*s **2018**, *36*, 575-587.
10. Portillo, M.C.; Moreno, O.P.; Mora-Ramírez, M.A.; Santiesteban, H.J.; Avendaño, C.B.; Bernal, Y.P. Optical and structural analysis of the charge transfer of Ce^{3+}+ e^{-}→Ce^{4+} ion in the cerium oxide (CeO_2), *Optik* **2021**, *248*, 168178.
11. Skorodumova, N.V.; Ahuja, R.; Simak, S.I.; Abrikosov, I.A.; Johansson, B.; Lundqvist, B.I. Electronic, bonding, and optical properties of CeO_2 and Ce_2O_3 from first principles. *Phys. Rev. B* **2001**, *64*, 115108.
12. Đorđevic, M.P.; Vukoje, I.; Lazic, V.; Đorđevic, V.; Sredojevic, D.; Dostanic, J.; Loncarevic, D.; Ahrenkiel, S.P.; Belic, M.R.; Nedeljkovic, J.M. Electronic structure of surface complexes between CeO_2 and benzene derivatives: A comparative experimental and DFT study. *Mater. Chem. Phy*. **2019**, *236*, 121816.
13. Marabelli, F.; Wachter, P. Covalent insulator CeO_2: Optical reflectivity measurements. *Phys. Rev. B* **1987**, *36*, 1238-1243.
14. Cresi, J.S.P.; Di Mario, L.; Catone, D.; Martelli, F.; Paladini, A.; Turchini, S.; D'Addato, S.; Luches, P.; O'Keeffe, P. Ultrafast formation of small polarons and the optical gap in CeO_2. *J. Phys. Chem. Lett.*, **2020**, *11*, 5686−5691.
15. Imhof, S.; Thränhardt, A. Phonon-assisted transitions and optical gain in indirect semiconductors. *Phys. Rev. B* **2010**, *82*, 085303.
16. Hassanien, A.S.; Akl, A.A. Effect of Se addition on optical and electrical properties of chalcogenide CdSSe thin films. *Superlattices Microstruct*. **2016,** *89*, 153-169.
17. Fifere, N.; Airinei, A.; Timpu, D.; Rotaru, A.; Sacarescu, L.; Ursu, L. New insights into structural and magnetic properties of of Ce doped ZnO nanoparticles. *J. Alloys Compd*. **2018**, *757*, 60–69.
18. Dragan, E.S.; Lazar, M.M.; Dinu, M.V.; Dascalu, I.A.; Nacu, I.; Verestiuc, L. Superelastic chitosan/laponite nanocomposite sponges with tunable functional properties as promising biomaterials for wound management. *ACS Appl. Bio Mater.* **2025**, *8*, 7699−7714.
